# A reference genome of Commelinales provides insights into the commelinids evolution and global spread of water hyacinth (*Pontederia crassipes*)

**DOI:** 10.1093/gigascience/giae006

**Published:** 2024-03-14

**Authors:** Yujie Huang, Longbiao Guo, Lingjuan Xie, Nianmin Shang, Dongya Wu, Chuyu Ye, Eduardo Carlos Rudell, Kazunori Okada, Qian-Hao Zhu, Beng-Kah Song, Daguang Cai, Aldo Merotto Junior, Lianyang Bai, Longjiang Fan

**Affiliations:** Institute of Crop Sciences & Institute of Bioinformatics, College of Agriculture and Biotechnology, Zhejiang University, Hangzhou 310058, China; Zhongyuan Institute of Zhejiang University, Zhengzhou 450000, China; State Key Laboratory of Rice Biology, China National Rice Research Institute, Hangzhou 310006, China; Institute of Crop Sciences & Institute of Bioinformatics, College of Agriculture and Biotechnology, Zhejiang University, Hangzhou 310058, China; Institute of Crop Sciences & Institute of Bioinformatics, College of Agriculture and Biotechnology, Zhejiang University, Hangzhou 310058, China; Institute of Crop Sciences & Institute of Bioinformatics, College of Agriculture and Biotechnology, Zhejiang University, Hangzhou 310058, China; Institute of Crop Sciences & Institute of Bioinformatics, College of Agriculture and Biotechnology, Zhejiang University, Hangzhou 310058, China; Department of Crop Sciences, Agricultural School, Federal University of Rio Grande do Sul, Porto Alegre, RS 68011, Brazil; Agro-Biotechnology Research Center (AgTECH), University of Tokyo, Tokyo 113-8657, Japan; CSIRO Agriculture and Food, Black Mountain Laboratories, Canberra, ACT 2601, Australia; School of Science, Monash University Malaysia, Bandar Sunway, Selangor 46150, Malaysia; Department of Molecular Phytopathology and Biotechnology, Christian Albrechts University of Kiel, Kiel D-24118, Germany; Department of Crop Sciences, Agricultural School, Federal University of Rio Grande do Sul, Porto Alegre, RS 68011, Brazil; Hunan Weed Science Key Laboratory, Hunan Academy of Agriculture Science, Changsha 410125, China; Institute of Crop Sciences & Institute of Bioinformatics, College of Agriculture and Biotechnology, Zhejiang University, Hangzhou 310058, China; Zhongyuan Institute of Zhejiang University, Zhengzhou 450000, China

**Keywords:** *Pontederia crassipes or Eichhornia crassipes*, Commelinales, reference genome, phylogeny of the commelinids, genetic diversity, karyotypes

## Abstract

Commelinales belongs to the commelinids clade, which also comprises Poales that includes the most important monocot species, such as rice, wheat, and maize. No reference genome of Commelinales is currently available. Water hyacinth (*Pontederia crassipes* or *Eichhornia crassipes*), a member of Commelinales, is one of the devastating aquatic weeds, although it is also grown as an ornamental and medical plant. Here, we present a chromosome-scale reference genome of the tetraploid water hyacinth with a total length of 1.22 Gb (over 95% of the estimated size) across 8 pseudochromosome pairs. With the representative genomes, we reconstructed a phylogeny of the commelinids, which supported Zingiberales and Commelinales being sister lineages of Arecales and shed lights on the controversial relationship of the orders. We also reconstructed ancestral karyotypes of the commelinids clade and confirmed the ancient commelinids genome having 8 chromosomes but not 5 as previously reported. Gene family analysis revealed contraction of disease-resistance genes during polyploidization of water hyacinth, likely a result of fitness requirement for its role as a weed. Genetic diversity analysis using 9 water hyacinth lines from 3 continents (South America, Asia, and Europe) revealed very closely related nuclear genomes and almost identical chloroplast genomes of the materials, as well as provided clues about the global dispersal of water hyacinth. The genomic resources of *P. crassipes* reported here contribute a crucial missing link of the commelinids species and offer novel insights into their phylogeny.

## Introduction

*Pontederia crassipes* (NCBI: txid44947, former name *Eichhornia crassipes*), commonly known as water hyacinth, belongs to Pontederiaceae of the Commelinales and is a perennial floating plant with light blue or purple flowers. *P. crassipes* is an allopolyploid with 32 chromosomes (2n = 4x = 32) [1]. Water hyacinth originated from the Amazon Basin, South America, and has spread to the tropics and subtropics since the 1800s to have a pan-tropical distribution across the world [2]. It is recognized as an exceedingly aggressive aquatic plant species that exhibits rapid growth and possesses the capacity for both sexual and asexual reproduction [3]. Although restricted to freshwater environments, it can effectively utilize nutrients, so it flourishes particularly in ecosystems with high nutrient loading, consequently outcompeting native plant species for space and sunlight [3–6]. As a result, it has been recognized by the International Union for Conservation of Nature as one of the 100 most invasive species and has been listed among the 10 most serious weed plants in the world [7, 8].

Commelinales is a branch of the commelinids clade, which also comprises Poales, Zingiberales, and Arecales. Many members of commelinids, such as rice, wheat, and maize, provide calorie-rich grains, livestock feed, and industrial raw materials [9–11]. A phylogenic tree of the commelinids has been constructed based on plastid genomes [12]. However, the controversy surrounding the phylogeny of commelinids, especially the placement of Poales and Commelinales, persists [12–14]. This uncertainty is attributed to discordance between nuclear and organellar phylogenies, which may arise from hybridization, incomplete lineage sorting, gene duplication, and gene loss [15–17]. Nuclear–plastid conflicts are prevalent at different taxonomic levels of angiosperms, such as the placement of the Celastrales, Malpighiales, and Oxalidales clade and the commelinids [13, 18, 19]. Many genomes of the economically important members of the commelinids have been sequenced, for example, the grasses (Poaceae), gingers and bananas (Zingiberales), and palms (Arecaceae). However, no reference genome within the Commelinales order has been generated up to now, which has hindered the elucidation of the phylogenetic puzzle of commelinids.

Here, we generated a chromosome-scale genome assembly of *P. crassipes*, investigated genome evolution of *P. crassipes* in relation to its related species to determine the phylogeny and ancestral karyotype of the commelinids, and further explored the genetic diversity and phylogenetic relationships of water hyacinth using materials collected from several countries.

## Results

### Genome assembly, phasing, and annotation

We sequenced an *P. crassipes* individual (Zijingang#1) collected from Hangzhou, China. The estimated genome size of *P. crassipes* was ∼1,058 Mb based on a *k*-mer survey using Illumina short reads (Supplementary Fig. S1A) and ∼1,278 Mb based on flow cytometry, consistent with its C-value of 1.28 pg/1C [20] (Supplementary Fig. S1B-D). The heterozygosity level of the *P. crassipes* genome was estimated to be 0.76%, and repetitive content accounts for 68.85% of the genome (Supplementary Fig. S1A). Based on 68-Gb (52×) HiFi reads with an average read length of 17.72 kb, a *de novo* assembly yielded a genome of 1.30 Gb, including 1,699 contigs with a contig N50 size of 39.5 Mb (Supplementary Table S1).

With the 130 Gb Hi-C data generated by this study, we assembled the genome of *P. crassipes* by anchoring 606 contigs to 16 superscaffolds (pseudochromosomes) with a total length of 1.22 Gb, representing 95.3% of the estimated size (Table 1). Attributed to the nature of tetraploid, the high collinearity between the 2 subgenomes brings challenges to assembly, which severely reduced the reliability of the regular ordering methods. Given that allopolyploids contain subgenome-specific sequences, we searched a subgenome-specific sequence (*k*-mer) and then clustered the specific sequences that differentiate homoeologous chromosomes, which enabled consistent partitioning of the genome into 2 subgenomes (Supplementary Fig. S2). Consequently, 16 superscaffolds were assigned to the 2 subgenomes, termed subA and subB. After phasing and ordering with directional interactions, we finally assembled the genome of *P. crassipes* with the size of subA and subB being 640.2 Mb and 577.6 Mb, respectively, and the size of pseudochromosomes ranged from 45.81 to 104.49 Mb (Table 1).

**Table 1:** Summary of *P. crassipes* plant materials collection, genome sequencing, and annotation by this study

Items	Data	
Reference genome		
Plant material	Zijingang#1 from Hangzhou, China	
Estimated genome size, Mb	1,278	
Sequencing platform (genome coverage)	PacBio HiFi (52×) + Illumina (61×) + HiC (100×)	
Assembly size, Mb	1,220	
Scaffold N50, Mb	77.2	
Number of genes annotated	65,299	
BUSCO assessment, %	95.2%	
Subgenome	SubA	SubB
Assembly size, Mb	640.2	577.6
Number of genes annotated	33,608	31,691
BUSCO assessment, %	88.3%	86.5%
Percentage of repeat elements, %	58.61%	52.92%
Population investigation		
Sequencing platform (genome coverage)	Illumina (36×)	
Number of collection locations	9	
Country sampled	Brazil (5), China (1), Malaysia (1), Germany (2)	

The quality of the assembly was validated through mapping 98.51% of the genomic short reads obtained by Illumina sequencing to the assembly. The long terminal repeat assembly index score was 11.78, indicating a reference quality comparable with those of Arabidopsis (TAIR10) and *Vitis vinifera* [21, 22]. We also estimated base-level accuracy and completeness of the genome and achieved a high assembly consensus quality value (42.0). High genomic synteny was observed between the *P. crassipes* assembly and other genomes within the commelinids clade (e.g., *Cocos nucifera*) (further details are available in the following sections). Taken together, the results suggested the reliability of the *P. crassipes* assembly.

A total of 65,299 genes were predicted in the *P. crassipes* assembly by applying a combination of homology, transcript-based and *ab initio* gene predictions approaches, after filtering out 732.02 Mb (56.09%) of repetitive sequences. Subsequently, we identified 33,608 and 31,691 genes in subgenomes A and B, respectively. BUSCO was used to assess the completeness of our genome annotation, which revealed that the gene set we annotated encompassed 1,536 (95.2%) of the 1,614 universal single-copy genes present in the Embryophyta lineage [23] (Supplementary Table S2).

### Phylogenetic position of *P. crassipes* based on single-copy genes

To resolve the phylogenetic position of Commelinales in the commelinids, we first constructed a phylogenetic tree using a concatenated sequence of 180 single-copy orthologs identified by OrthoFinder [24] of the water hyacinth genome and 7 other representative members with high-quality genomes, using *Acorus tatarinowii* as the outgroup (Supplementary Fig. S3A). The phylogenetic tree revealed that Zingiberales and Commelinales were sister lineages of Arecales, and Poales is located in the out node, which supports the previous phylogenetic studies by Cheng et al. [25] and Wang et al. [ 26].

To validate the stability of the phylogenetic tree, we reconstructed a maximum likelihood phylogeny by utilizing a concatenated matrix comprising 180 single-copy orthologs from the 9 genomes. A coalescent-based phylogeny was also generated through integration of the single-copy gene trees (Supplementary Fig. S3B, C). The topologies of both the coalescent and concatenate trees supported the aforementioned ortholog-based tree. Strong robustness was evident at each node (Supplementary Fig. S3B) within the coalescent tree. At the same time, the outcomes were also in accordance with a consensus tree generated using DensiTree [27] (Supplementary Fig. S3D).

**Figure 1:**
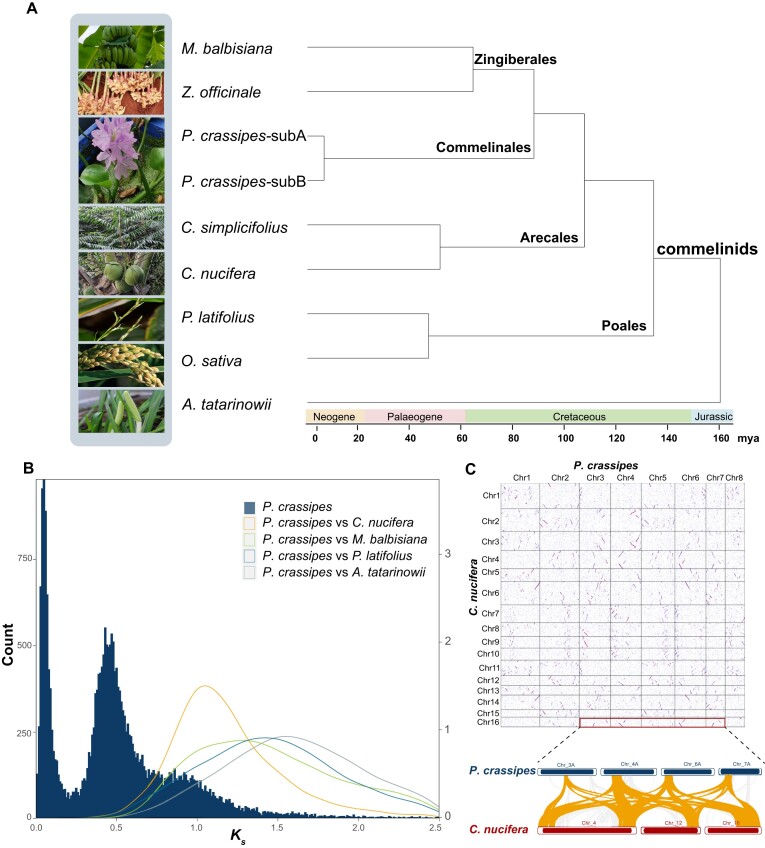
Phylogeny and evolution of the *P. crassipes* genome. (A) Single-copy gene-based ultrametric phylogenetic tree and divergence times of *P. crassipes* and other representative species of the commelinids with *A. tatarinowii* as an outgroup. (B) Distribution of synonymous substitution per site (*K_S_*) of paralog genes in collinear regions of *P. crassipes* and orthologous genes between *P. crassipes* and other members of the commelinids (*C. nucifera, C. simplicifolius, Z. officinale, M. balbisiana, P. latifolius*, and *A. tatarinowii*). (C) Dot plots showing the conserved genomic synteny between *P. crassipes* and *C. nucifera*. An example of conserved synteny region originating from the τ WGD event is marked with a rectangle.

To date evolutionary events, we reconstructed a time-calibrated phylogenetic tree combined with fossil calibration time (Fig. 1A). The analysis showed an early origin of commelinids in Jurassic, ∼160 million years ago (mya) (136.6–201.3 under 95% confidence interval) (Fig. 1A). Commelinales arose at ∼87.7 mya (82.1–93.2), and the divergence time between subgenomes A and B of *P. crassipes* was dated at ∼6.4 mya (4.4–9.0).

### Whole-genome duplications of *P. crassipes*

Whole-genome duplications (WGDs) cause rapid genome reorganization and structural variations to produce new chromosomal karyotypes [28, 29]. The analysis of genomic synteny showed excellent collinearity within the *P. crassipes* genome, which suggested recent genomic duplication events (Supplementary Fig. S4). Based on the syntenic blocks, we clustered the pseudochromosomes into ancestral chromosomes as A1 (Chr1A–4A), A2 (Chr5A–8A), B1 (Chr1B–4B), and B2 (Chr5B–8B). To confirm potential WGD events in the water hyacinth genome and estimate divergence time, we extracted syntenic gene pairs within the *P. crassipes* genome and their orthologs in 4 representative species of the commelinids (*A. tatarinowii, Musa balbisiana, C. nucifera*, and *Pharus latifolius*). The distribution of synonymous substitutions per site (*K_S_*) indicated that at least 3 rounds of WGDs happened during *P. crassipes* evolution, consistent with the above synteny analysis results (Fig. 1B). However, the estimated divergence of water hyacinth and palms of Arecaceae (*K_S_* = 1.04) occurred after divergence from Zingiberales (*K_S_* = 1.17) according to the *K_S_* peaks, which conflicts with the phylogenetic tree (Fig. 1A). The stronger collinearity between water hyacinth and palms seemed to support the result of *K_s_* distribution (Fig. 1C).

The conflict between the *K_S_* inference and phylogenetic analysis might be triggered by several factors, such as different substitution rates or structural genomic rearrangement rates [30, 31]. To test this hypothesis, we inferred the substitution rate in each branch with Bayesian methods implemented in BEAST2 [32]. Concordant with the hypothesis, the estimated substitution rate in the palm (0.67) was significantly less than that in the ginger (1.18), indicating that the evolutionary rate variation across the taxa caused the bias of the *K_S_* distribution.

We further extracted paralogs present in the genomes derived from the WGDs, aiming to elucidate the orders and dates of the WGD events that transpired during the evolution of water hyacinth. Two prominent peaks of the *K_S_* distribution of water hyacinth (Fig. 1B) suggested 2 relatively recent WGD events. These events encompassed the most recent tetraploidization event and a duplication event specific to the Commelinales lineage. Water hyacinth shared an ancient WGD with other commelinids, which has been recognized as the τ WGD event [26] (Supplementary Fig. S5). To confirm the ancient duplication process, we estimated the copy number in collinear regions between the water hyacinth and coconut genomes and found that some genomic regions indeed shared 4 corresponding copies in the 2 genomes (Fig. 1C). A case with the detailed genomic synteny between the 2 genomes (water hyacinth Chr3, Chr4, Chr6, Chr7 vs. coconuts Chr4, Chr12, Chr16) is shown in Fig. 1C. Following the estimated time of *τ* WGD (129–146 mya) based on the coconut genome [26], the tetraploidization event of water hyacinth was estimated to occur approximately 8–10 mya and the lineage-specific duplication at 67–76 mya, which all were comparable to the phylogenetic estimates (Fig. 1A). Differentiated transposable element (TE) contents were observed in 2 subgenomes of water hyacinth, with a divergence rate ranging from 2% to 8% (subA) and 16% to 22% (subB). These differences resulted in the formation of a distinctive “bubble” peak within the TE profile, indicating a WGD pattern similar to that observed in the analysis of collinear paralogous pairs (Supplementary Fig. S6).

### Mass loss of disease-resistance genes in the *P. crassipes* genome

**Figure 2:**
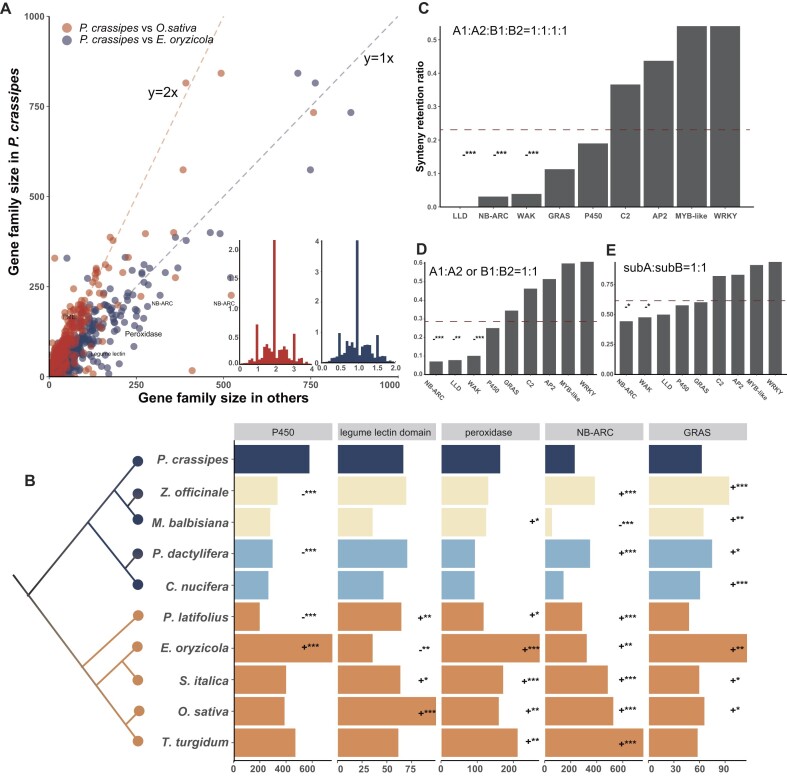
Changes of gene family size during genome polyploidization of *P. crassipes*. (A) Dot matrix plot and distribution of fold changes of gene family sizes in *P. crassipes* compared with diploid *O. sativa* and tetraploid *E. oryzicola*. Regarding the distribution of gene family sizes (subfigures at lower right corner), the highest percentage was observed in the gene families of *P. crassipes* that were 2 times bigger in size than those of *O. sativa* (left) and the same size as those of *E. oryzicola* (right). (B) Comparison of disease resistance–related gene family sizes between *P. crassipes* and other commelinids species. + and − indicate increase and decrease in size, respectively, relative to *P. crassipe*s. ∗*P* < 0.01, ∗∗*P* < 0.001, ∗∗∗*P* < 0.0001, Fisher’s exact test. (C–E) Synteny retention ratio of different paralogous pairs in 10 gene families after polyploidization of *P. crassipes*. The graphs show the percentage of retained gene pairs that experienced the 2 polyploidization events (C, A1:A2:B1:B2 = 1:1:1:1), 1 of the 2 events (D, A1:A2 or B1:B2 = 1:1), or 2 subgenomes (E, subA:subB = 1:1). The dashed lines represent the average retention ratio of genes across the genome. LLD: legume lectin domain.

To estimate gene loss and gain during polyploidization, gene family sizes were determined by identifying protein domains in *P. crassipes* and other representative genomes. We first compared gene family sizes between tetraploid *P. crassipes* and the diploid *Oryza sativa* genome using a dot matrix plot (Fig. 2A). The results showed that the size of the majority of gene families in *P. crassipes* was almost 2 times higher than those in *O. sativa*, consistent with their ploidy. The analysis also revealed that the size of several gene families (predominantly associated with disease resistance) in *P. crassipes* was significantly smaller than expected, for example, genes encoding NB-ARC (226 in *P. crassipes* vs. 522 and 480 in *O. sativa* and another diploid grass *Setaria italica*, respectively), GRAS (62 vs. 65 and 59, respectively), peroxidase (162 vs. 158 and 170, respectively), and legume lectin (66 vs. 99 and 63, respectively) (Fig. 2E) [33–37]. We also compared the gene family size between water hyacinth and 2 other species of the grass family, tetraploid weed *Echinochloa oryzicola* (Fig. 2A) and crop durum wheat (*Triticum turgidum*), and found the same trend (Fig. 2B). For example, the number of NB-ARC genes in durum wheat (753) and *E. oryzicola* (318) was higher than in water hyacinth (226) (*P* < 0.001, Fisher’s exact test). The results suggested a contraction of disease-resistance genes in the *P. crassipes* genome, consistent with the phenomenon observed in the *Echinochloa* weeds [38].

To estimate the loss/gain of disease-resistance genes during duplication, we calculated synteny retention ratios of collinear gene pairs in the water hyacinth genome by estimating the percentage of the retained gene pairs that experienced the 2 polyploidization events (A1:A2:B1:B2 = 1:1:1:1) and 1 of the 2 events (A1:A2 or B1:B2 = 1:1), as well as between the 2 subgenomes (subA:subB = 1:1) (Fig. 2C–E). Across the genome, while 23.2% of genes fit the 1:1:1:1 (A1:A2:B1:B2) synteny retention ratio (Fig. 2C), the synteny retention ratio of the NB-ARC family genes (3.8%) was significantly lower (*P* < 0.0001, Fisher’s exact test); similarly, a significantly low synteny retention ratio (3.1%; *P* < 0.0001) was also evident for another well-known disease-resistance gene family, the wall-associated receptor kinases [39]. To identify the conversation pattern of the gene families after polyploidization events, we also compared synteny retention ratios of collinear gene pairs that originated from different events (i.e., A1:A2 or B1:B2 = 1:1). The results illustrated that genes encoding NB-ARC (7%, *P* < 0.0001; 44%, *P* < 0.01) and wall-associated receptor kinases (10.1%, *P* < 0.0001; 47%, *P* < 0.01) suffered significant loss after polyploidization (Fig. 2D, E).

The same bioinformatics pipeline was used to compare the patterns of gene retention and loss in the commelinids for several other gene families. A higher number of P450 genes (574) was observed in *P. crassipes* compared with other species, possibly related to its capacity of survival in the severely polluted conditions (Fig. 2B). In Arecales and Zingiberales, the increased number of GRAS genes implied a reduction of the gene family during divergence of *P. crassipes* (Fig. 2B). Consistent with the findings from previous studies [40–42], we observed a significant increase of disease-resistance genes in crops, including genes encoding legume lectin, peroxidase, and NB-ARC.

### Ancestral karyotype evolution of the commelinids

**Figure 3:**
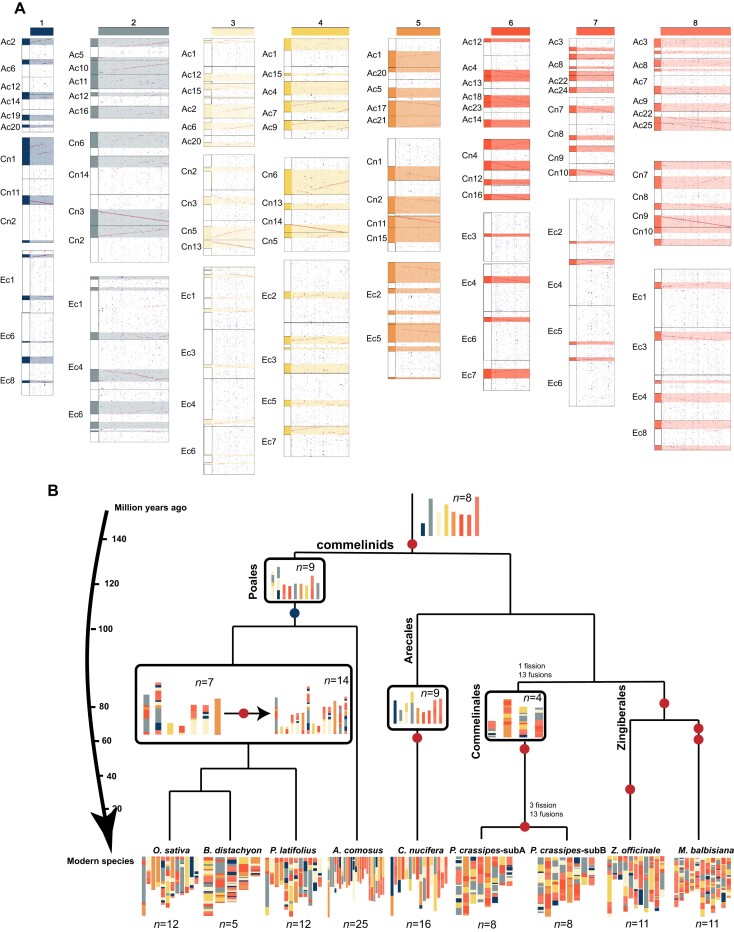
Inference of proto-chromosomes and ancestral karyotypes of the commelinids. (A) Identification of proto-chromosomes based on synteny regions among extant chromosomes. Alignments between proto- and extant chromosomes shown in different colors indicate the different origination from the proto-chromosomes. Ac: *A. comosus*; Cn: *C. nucifera*; Pc: *P. crassipes*. (B) Reconstruction of ancestral karyotypes and their phylogeny of the commelinids. Ancestral chromosomes at specific evolutionary nodes were inferred and denoted with different colors. Whole-genome duplication and triplication events are shown in red and blue circles, respectively.

Being a key phylogenetic branch within the commelinids clade, the high-quality reference genome of Commelinales generated in this study provides an opportunity to reconstruct the ancestral karyotype of the commelinids. We therefore compared 7 representative species with well-assembled genomes with *P. crassipes* (Fig. 3A). By inferring intergenomic gene collinearity, we mapped the 7 genomes onto *P. crassipes* and estimated the ratio of the best-matched orthologous regions between *P. crassipes* and *C. nucifera* (Arecaceae), *Ananas comosus* (Poaceae), and *Zingiber officinale* (Zingiberales), being 4:2, 4:3, and 4:4, respectively, a result consistent with the WGD times experienced by the species (Supplementary Fig. S7A). Based on the gene collinearity of the 4 genomes (Fig. 3A, Supplementary Fig. S13a–c), we constructed an ancestral karyotype with 8 proto-chromosomes shared by the commelinids (Fig. 3B). Accordingly, we also reconstructed the ancestral karyotypes of 4 other species, *O. sativa, M. balbisiana, Brachypodium distachyon*, and *P. latifolius*.

The reconstruction results clearly showed frequent chromosomal rearrangements in *P. crassipes* and genome structure changes in Zingiberales (Fig. 3B). A close check of shared collinearity between extant plant chromosomes identified the origin of certain extant chromosomes, thereby revealing their antiquity. For example, the region originated from τ WGD, located in chromosome 6 of *C. nucifera* and chromosome 1 of *P. crassipes* (Fig. 3A). From the deduced ancestral state, Commelinales proto-chromosomes have been shaped through *τ* WGD followed by 1 fission and 13 fusions to reach an *n* = 4 intermediate state. Then 3 fissions and 13 fusions accounted for the transition between the *n* = 4 intermediate state and the modern genome structure of 8 chromosomes in subgenomes A and B of *P. crassipes*. The fewest chromosomal rearrangements were observed in *C. nucifera*, consistent with its low nucleotide substitution rate, while Zingiberales underwent similar massive chromosomal rearrangements.

### Genetic diversity of *P. crassipes*

**Figure 4:**
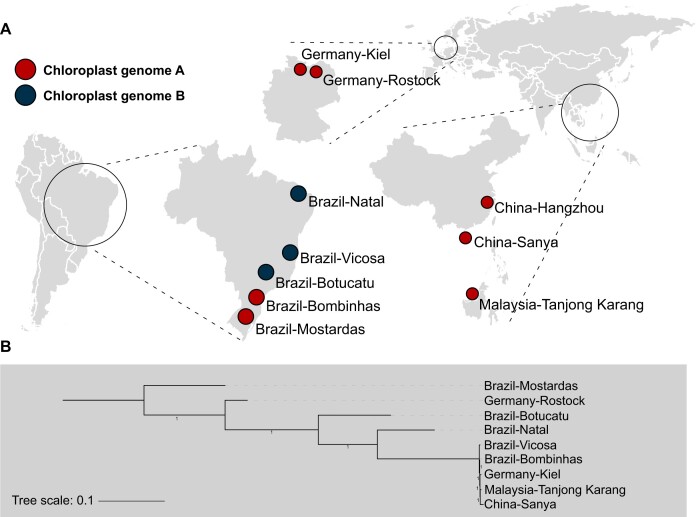
Genetic diversity and phylogeny of global *P. crassipes*. (A) The collection locations of the 10 water hyacinth lines used in this study are indicated by circles and the chloroplast genomes (A and B) are labeled with two different colors. (B) A phylogenetic tree of the 9 lines built based on their nuclear genomic SNPs relative to the reference Zijingang genome.

To estimate genetic diversity of global water hyacinth, we collected an additional 9 lines from South America (Brazil), Asia (China and Malaysia), and Europe (Germany) (Fig. 4) and sequenced them with an average of 36× genomic coverage. Based on the single-nucleotide polymorphisms (SNPs) among the 9 genomes and the *P. crassipes* reference genome (Zijingang#1), we found a relatively low genetic diversity (*π* = 1.44 × 10^−3^) of the global water hyacinth, compared to sorghum (3.05 × 10^−3^) and other crops [43]. Based on principal component analysis (PCA), the water hyacinth native to Brazil (5 lines from different locations) seemed to have a relatively higher diversity than those from other countries (Malaysia, China, and Germany) (Supplementary Fig. S8), indicating a tendency of a more divergent genetic diversity of the species in the area of its origin [44].

The phylogenetic tree of the global water hyacinth was consistent with the PCA results (Fig. 4), in which the Brazil lines embraced the 3 lines from the 3 other countries. Of all 5 non-Brazilian lines, except for one of the lines from Germany (Germany_Rostock), the other 4 lines (including the Zijingang#1 line) had almost the same nuclear genome as the 2 Brazilian lines (Brazil_Vicosa and Brazil_Bombinhas). The chloroplast genomes of all 10 lines were further assembled, and surprisingly, only 2 chloroplast genomes (named chloroplast genomes A and B) of water hyacinth, which were nearly identical and differed by only a 1-bp indel (Fig. 4A), were achieved. In Brazil, the chloroplast genomes A and B were observed in lines from the southern and northern areas, respectively, while all water hyacinth lines from other countries had genome A. Taken together, these results support Brazil as one of the origins of water hyacinth and suggest a global spread potentially by 1 or 2 genotypes.

## Discussion

At present, all of the major commelinids crops (e.g., rice, wheat, and maize) [45–47] and other important economic crops of the clade such as pineapple and bananas [45, 48, 49] have had their genomes sequenced. However, the Commelinales order, an important phylogenetic node of the commelinids, still lacks a reference genome until now. Here we generated a high-quality reference genome of *P. crassipes*, representing the first genome of the Commelinales order. The availability of the genome provides a crucial missing link among different orders of the commelinids clade and is anticipated to facilitate studies of genome evolution.

The analysis of the ancient karyotype of the commelinids provides clear evidence for the clade having 8 proto-chromosomes. While the result differs from the result of 5 proto-chromosomes reported by other studies [50, 51], it is in line with the result based on a study of coconuts [26]. Apparently, the lack of high-quality genomes of representative species of crucial nodes of a phylogenetic tree hinders the inference of an evolutionary framework [52]. With the continuously increasing number of high-quality genomes, particularly the genomes filling the missing links, such as the water hyacinth genome generated in this study, gene collinearity and syntenic blocks between different species of the commelinids clade can be more clearly defined and characterized, shedding lights on the plasticity of the commelinids genomes and their evolutionary trajectories.

Water hyacinth seemed to have experienced a significant reduction in disease-resistance genes (such as NB-ARC, GRAS, peroxidase, and legume lectin) during its evolutionary history. This could potentially be linked to fitness costs associated with allocating energy toward growth and reproduction processes [53–55]. Emerging data demonstrate that the growth–defense trade-offs allow plants to adjust growth and defense based on external conditions [56]. The phenomenon of shrinking of disease-resistance genes has also been observed in other noxious weeds [38, 53–55]. It is reasonable to assume, therefore, that the loss of the disease-resistance gene in the *P. crassipes* genome could be a result of natural selection to maximize and accelerate the growth and reproduction of *P. crassipes*. However, it is also possible that fewer disease-resistance genes evolved during its evolutionary history due to lower disease pressure in the surrounding environment (water) where *P. crassipes* grows. Significant contractions in certain disease-resistance gene families imply stronger competitiveness and invasiveness of *P. crassipes*. While strong disease resistance is an important agronomic trait for crops, rapid growth and extensive reproduction may be necessary for weediness and invasiveness in general. Further investigation of the underlying mechanisms, such as fitness costs in weeds, will thus contribute to a better understanding of their invasive strategy and could potentially be used to develop effective weed management strategies.

This study revealed both identical nuclear and chloroplast genomes between some of the Brazilian water hyacinth and all the water hyacinth from other countries (except the German Rostock line), indicating the spread of a limited genotype of water hyacinth from South America, where it has the highest genetic diversity. The genetic uniformity has been observed in the global spread of water hyacinth and other invasive species [7, 57]. Bombinhas is a city in the southern region of Brazil, located in close proximity to the Itajaí Port, the sixth largest port in Brazil, established in the early 1860s. Given the strategic location of the Itajaí Port on the South American East Coast, there is a possibility that the early invasion abroad of water hyacinth could have been facilitated by transportation/immigration from the Itajaí Port, which was not mentioned in Brazil’s history. Although the Rostock line may indicate additional global dispersal of water hyacinth, our results indicated that the available non-Brazilian water hyacinth may have originated from Brazil.

## Materials and Methods

### Materials collection and sequencing

A wild *P. crassipes* plant (Zijingang#1) collected from Zijingang Campus of Zhejiang University, Hangzhou, China, was used in construction of the reference genome. The additional 9 lines of *P. crassipes* were collected globally for phylognetic analysis, with their detailed information available in Supplementary Table S3. Genomic DNA of *P. crassipes* was extracted from young leaves using the CTAB method for sequencing library construction. Following the standard protocols of the Pacific Biosciences, DNA libraries for single-molecule real-time PacBio genome sequencing were constructed and circular consensus sequencing was performed on the PacBio Sequel2 platform (RRID:SCR_017990) for high-fidelity (HiFi) reads. Short-read libraries of *P. crassipes* were constructed according to Illumina’s standard protocol, and paired-end reads (2 × 150 bp) were sequenced on an Illumina HiSeq X Ten platform (RRID:SCR_020131). With default parameters, raw PacBio subreads were filtered and corrected using the pbccs pipeline.

A Hi-C library was constructed using fresh young leaves of *P. crassipes*, which were fixed in 1% formaldehyde for crosslinking. Cells were lysed using a Dounce homogenizer and digested using the *Hin*d III restriction enzyme. The DNA ends were filled and labeled with biotin and the filled-in *Hin*d III sites were ligated to form *Nhe* I sites. Complexes with the biotin-labeled ligation products were purified and sheared, and the biotinylated Hi-C ligation products were pulled down and used to construct Illumina sequencing libraries [58].

### Genome assembly

The HiFi reads were subjected to hifiasm (RRID:SCR_021069) [59] for *de novo* assembly in default mode. After mapping the long subreads to the initial assembly with minimap2 (RRID:SCR_018550) [60], racon [61] was used in 3 rounds of correction with default parameters. Based on the subassembly, clean Hi-C reads were analyzed and 3D-DNA [62] was used to scaffold contigs into pseudochromosomes followed by being manually corrected with Juicer (RRID:SCR_017226) [63].

The above genome assembly was subjected to SubPhaser [64] to search the subgenome-specific sequence (*k*-mer), and then homoeologous chromosomes were assigned into 2 subgenomes (Supplementary Fig. S2). Based on the coverage depth of the short reads against the assembly, we manually corrected some errors with discrete chromatin interaction patterns. The assembled genome was subjected to BUSCO v5.5.0 (RRID:SCR_015008) [23] with embryophyta_odb 10 to evaluate the completeness of the genome.

### Genome annotation

Repeat families were first identified *de novo* and classified initially using RepeatModeler v1.0.10 (RRID:SCR_015027) [65]. The repeat library by RepeatModeler was analyzed with RepeatMasker v4.0.7 (RRID:SCR_012954) [65] for the whole-genome repeat annotation.

A hybrid strategy integrating *ab initio* predictions by Fgenesh [66] and AUGUSTUS v3.2.2 (RRID:SCR_017555) [67], homolog evidence-based prediction, and transcript-assisted predictions was applied for gene prediction. EVidenceModeler v1.1.1 (RRID:SCR_014659) [68] was used to integrate the gene models predicted by the above approaches to obtain a nonredundant consensus gene set. Gene models were identified as those supported by homologous genes or transcript evidence or by at least 2 *ab initio* methods. High-confidence gene models were further filtered to remove short gene models (<50 amino acids) and gene models with homology to sequences in the Repbase (RRID:SCR_021169) (E value ≤1 × 10^−5^, identity ≥30%, coverage ≥25%). Functional annotations of protein-coding genes were conducted based on Pfam protein domains using InterProScan v5.24–63.0 (RRID:SCR_005829) [69].

Tandem repeats were identified with Satellite Repeat Finder [70], and 1 type of centromere sequences was found. To precisely annotate the location of the centromeric monomers *CEN148*, we calculated peak values in the windows of the divided genome and merged the windows with the same kind of monomers.

### Divergence time estimation

Phylogenetic trees for *P. crassipes* and 7 other species (*M. balbisiana* [71], *Z. officinale* [25], *Calamus simplicifolius* [72], *C. nucifera* [26], *P. latifolius* [73], *O. sativa* [74], and *A. tatarinowii* [51]) were built with FastTree [75] using 180 shared single-copy genes identified by OrthoFinder [24] and visualized in iTOL (itol.embl.de) [76]. The phylogenetic relationship was further checked by IQ-TREE 2 (RRID:SCR_017254) [77] with concatenated- and coalescent-based input data. The substitution rate in different branches was inferred in BEAST2 (RRID:SCR_017307) [32]. The species tree rooted with *A. tatarinowii* was used as an input to build an ultrametric tree by the MCMCTree program in PAML (RRID:SCR_014932) [78], whereas secondary calibration was set to *A. tatarinowii*–*O. sativa* (133.0–139.1 mya) derived from the Timetree database [79]. TE divergence was assessed by PercDivs (percentage of substitutions in the matching region compared with the consensus) calculated in RepeatMasker. TE sequence divergence between 2 subgenomes of tetraploid *P. crassipes* displaying a high degree of overlap suggested the consistency of the TE evolutionary rate in the 2 subgenomes (Supplementary Fig. S6). The nonoverlapping segregation region represents the period between the divergence of diploid progenitors and the merging of their genomes into a tetraploid genome [80].

### Genome polyploidization analysis

We selected 4 representative species, including *M. balbisiana, C. simplicifolius, P. latifolius*, and *A. tatarinowii*, for comparative genomics analysis with *E. crassipes*, aiming to investigate the polyploidization event(s) that occurred and whether they were shared or not, as well as infer the evolutionary trajectories that led to the formation of current chromosomes. We first aligned protein sequences manually among species or subgenomes. WGDI (Whole-Genome Duplication Integrated analysis) was used to identify collinear blocks, which are the genomic regions containing collinear genes according to the combined information of gene similarity and gene order, within and between each genome [81]. The maximum gap allowed between collinear genes on a chromosome was set to 50 intervening or noncollinear genes. To help date evolutionary events and identify collinear genes produced by different events, polyploidization, or speciation, *K_S_* between collinear genes was estimated using KaKs_calculator with the NG model [82]. Given the possible effects of diverse nucleotide substitution among different lineages for phylogeny estimation, shared polyploidization between water hyacinth and coconut was recognized as an anchor to date duplication events that occurred in water hyacinth.

### Analysis of ancestral karyotypes and chromosome evolutionary trajectories

To investigate the chromosome evolution of commelinid genomes, we selected representative species (Fig. 3B) from 4 orders with chromosome-level genome assembly. We identified homologous proteins between extant genomes and the reconstructed commelinid karyotypes and then used WGDI to detect syntenic blocks as described above [81]. Then, dot plots were created to show synteny, and the chromosomal rearrangements were reconstructed.

### Gene family identification

InterProScan (version 5.24–63.0) [69] was used to identify Pfam protein domains, which were used to identify gene families. Besides the *P. crassipes* genes annotated in this study, protein domains were also identified for the genes of *P. latifolius* [73], *S. italica* (v2.0) [83], *O. sativa* [74], *T. turgidum* [46], *E. oryzicola* [84], *M. balbisiana* [71], *Z. officinale* [25], *Phoenix dactylifera* [85], and *C. nucifera* [26].

### Resequencing and variant calls

For resequencing, short-read libraries of additional lines of *P. crassipes* collected globally were sequenced on an Illumina HiSeq X Ten platform (Supplementary Table S3). Raw data were first filtered by the NGSQC Toolkit (v2.3.348) [86]. Clean paired-end reads of each accession were then aligned to *P. crassipes* using Bowtie2 with default parameters. A custom pipeline [87] was used in calling and filtering variants. Low-quality variants were further removed with minor allele frequency <0.01 and missing rate >30%.

### Phylogenetic analysis and PCA

The phylogenetic analysis was performed on the full set of all 9 water hyacinth lines. A phylogeny tree was constructed based on 6.5 million SNPs using FastTree (RRID:SCR_015501) [75] and visualized in iTOL (RRID:SCR_018174) [76]. All SNPs with lines were analyzed using the R package SNPRelate to conduct PCA [88].

### Chloroplast genome assembly and annotation

The clean data of Illumina sequencing reads of all 9 lines were applied in *de novo* assembly by GetOrganelle (RRID:SCR_022963) [89]. Genome annotation was performed by the GeSeq (RRID:SCR_017336) online [90]. A custom script was used to filter out duplicate annotated genes. Multiple sequence alignment of chloroplast genomes was performed with MAFFT (Multiple Alignment based on Fast Fourier Transform) (RRID:SCR_011811) [91].

## Supplementary Material

giae006_GIGA-D-23-00274_Original_Submission

giae006_GIGA-D-23-00274_Revision_1

giae006_Response_to_Reviewer_Comments_Original_Submission

giae006_Reviewer_1_Report_Original_Submission Eric Schranz -- 10/29/2023 Reviewed

giae006_Reviewer_1_Report_Revision_1 Eric Schranz -- 12/22/2023 Reviewed

giae006_Reviewer_2_Report_Original_Submission Eric Patterson -- 11/1/2023 Reviewed

giae006_Reviewer_2_Report_Revision_1 Eric Patterson -- 12/22/2023 Reviewed

giae006_Supplemental_Files

## Data Availability

The genomic sequence and RNA-seq data of *P. crassipes* generated by this study were deposited into the NGDC (National Genomics Data Center) database under the accession number PRJCA020146 and European Nucleotide Archive (ENA) BioProject: PRJNA1062020. The assembled chloroplast genome sequences and annotation information have been submitted in NGDC under accession numbers C_AA041877.1, C_AA041878.1, and C_AA041879.1. All additional supporting data are available in the *GigaScience* repository, GigaDB [92].

## Additional Files

**Supplementary Fig. S1**. Genome size estimated based on *k*-mer analysis and flow cytometry analysis. A, *k*-mer analysis result with *k* = 17. B,C,D, three duplication of flow cytometry analysis to estimate the genome size. Rice was utilized as reference, and genome size of *E. crassipes* was calculate based on the ratio of the peaks.

**Supplementary Fig. S2**. Phased subgenomes of *E. crassipes* based on subgenome-specific *k*-mers. Rings from outer to inner was (1) Subgenome assignments of *E. crassipes*. (2) Significant enrichment of SubA and SubB specific *k*-mers. (3) Normalized proportion of SubA and SubB specific *k*-mers. (4-6) Density distribution (count) of each subgenome-specific *k*-mer set. (7) Density distribution (count) of subgenome-specific LTR-RTs and other LTR-RTs (the most outer, in grey color). (8) Homoeologous blocks of each homoeologous chromosome set.

**Supplementary Fig. S3**. Phylogeny relationship in the commlinids. A, a phylogenetic tree using 180 single-copy orthologs of the water hyacinth genome and other seven representative members with FastTree. B, A coalescent-based phylogeny generated through integration of the single-copy gene trees. C, a concatenate-based phylogeny generated through integration of the single-copy gene alignment. D, a consensus tree generated using DensiTree.

**Supplementary Fig. S4**. Synteny region inner *E. crassipes* genome. Dot plot was generate with synteny block was designated to harbor at least five consecutive gene pairs.

**Supplementary Fig. S5**. Distribution of synonymous substitution per site (*Ks*) of paralogue genes in collinear regions of *E. crassipes* and *C. nucifera*. The shared ancient duplication event was labeled with red.

**Supplementary Fig. S6**. Distribution of transposable element (TE) in different subgenomes of *E. crassipes*.

**Supplementary Fig. S7**. Duplication events and synteny region between *E. crassipes* and *A. comosus, Z. officinale, C. nucifera* chromosomes. A, distribution of synonymous substitution per site (*Ks*) of paralogue genes in collinear regions among *A. tatarinowii, O. sativa, C. nucifera, E. crassipes, M. balbisiana* and *Z. officinale*. B, dot plot of synteny block between *E. crassipes* and *C. nucifera*. C, dot plot of synteny block between *E. crassipes* and *A. comosus*, D, dot plot of synteny block between *E. crassipes* and *Z. officinale*.

**Supplementary Fig. S8**. Principle component analysis (PCA) of nine lines collected from South America (Brazil), Asia (China and Malaysia) and Europe (Germany). The water hyacinth native to Brazil (five lines from different locations) seemed to have a relatively higher diversity than those from other countries (Malaysia, China and Germany).

## Abbreviations

BUSCO: Benchmarking Universal Single-Copy Orthologs; HiFi: high fidelity; LLD: legume lectin domain; mya: million years ago; PCA: principal component analysis; SNP: single-nucleotide polymorphism; TE: transposable element; WGD: whole-genome duplication; MAFFT: multiple sequence alignment based on fast fourier transform; WGDI: whole-genome duplication integrated analysis.
