# Supplementary material for: A reference genome of Commelinales provides insights into the commelinids evolution and global spread of water hyacinth (Pontederia crassipes)
Source: Gigascience. 2024 Mar 14;13:giae006. doi: 10.1093/gigascience/giae006 (PMC10938897; doi:10.1093/gigascience/giae006)
Supplement: giae006_GIGA-D-23-00274_Revision_1 [file giae006_giga-d-23-00274_revision_1.pdf]

GigaScience

A reference genome of Commelinales provides insights into the commelinids evolution and global spread of water hyacinth (Pontederia crassipes)

--Manuscript Draft--

Manuscript Number:	GIGA-D-23-00274R1
Full Title:	A reference genome of Commelinales provides insights into the commelinids evolution and global spread of water hyacinth (Pontederia crassipes)
Article Type:	Research
Funding Information:	Key Technologies Research and Development Program (SQ2022YFD1400042) — Not applicable
Abstract:	Commelinales belongs to the commelinids clade which also comprises Poales that includes the most important monocot species, such as rice, wheat, and maize. No reference genome of Commelinales is current available. Water hyacinth (Pontederia crassipes or Eichhornia crassipes), a member of Commelinales, is one of the devastating aquatic weeds although it is also grown as an ornamental and medical plant. Here, we present a chromosome-scale reference genome of the tetraploid water hyacinth with a total length of 1.22 Gb (over 95% of the estimated size) across eight pseudochromosome pairs. With the representative genomes, we reconstructed phylogeny of the commelinids, which supported Zingiberales and Commelinales being sister lineages of Arecales and shed lights on the controversial relationship of the orders. We also reconstructed ancestral karyotypes of the commelinids clade and confirmed the ancient commelinids genome having eight chromosomes but not five as previously reported. Gene family analysis revealed contraction of disease-resistance genes during polyploidization of water hyacinth, likely a result of fitness requirement for its role as a weed. Genetic diversity analysis using nine water hyacinth lines from three continents (South America, Asia and Europe) revealed very closely related nuclear genomes and almost identical chloroplast genomes of the materials, and provided clues about the global dispersal of water hyacinth. The genomic resources of P. crassipes reported here contribute a crucial missing link of the commilinids species and offer novel insights into their phylogeny.
Corresponding Author:	Longjiang Fan<br>Zhejjiang University<br>Hangzhou, Zhejiang CHINA
Corresponding Author Secondary Information:	
Corresponding Author's Institution:	Zhejjiang University
Corresponding Author's Secondary Institution:	
First Author:	Yujie Huang
First Author Secondary Information:	
Order of Authors:	Yujie Huang
	longbiao Guo
	lingjuan Xie
	Nianmin Shang
	Dongya Wu
	Chuyu Ye
	Eduardo Carlos Rudell
	Kazunori Okada
	Qian-hao Zhu

Powered by Editorial Manager® and ProduXion Manager® from Aries Systems Corporation

	Beng-Kah Song
	Daguang Cai
	Aldo Merotto Junior
	Lianyang Bai
	Longjiang Fan
**Order of Authors Secondary Information:**	
**Response to Reviewers:**	MS# GIGA-D-23-00274<br><br>TITLE: A reference genome of Commelinales provides insights into the commelinids evolution and global spread of water hyacinth (Pontederia crassipes)<br><br><br>Dear Editor in Chief:\\<br><br>Thank you very much for giving us an opportunity to revise our manuscript. We have carefully considered all of the comments and concerns raised by the two reviewers. Below you will find a point-by-point responses to the Reviewers' comments. We hope that we have fully addressed your and the Reviewers' concerns and that the substantially revised version of the manuscript is now suitable for publication in GigaScience.<br>Thank you for your time and effort on handling our manuscript!<br><br>Sincerely,<br><br>Longjiang Fan<br><br>...<br>Reviewer #1:<br>[The authors used 180 genes to infer the phylogeny of eight species, but the information about alignment, model test, bootstrap etc. are missing. It's not clear how did the authors generate the "orthologs-based tree". Did the author take it from OrthoFinder? Then the author built coalescent and concatenate trees and compared them with "orthologs-based tree". In general, both coalescent and concatenate trees are also orthologs-based tree. It seems that the authors compared the trees generated by different ML-based software (IQtree and RaxML) using the same dataset (180 orthologs). This doesn't say much about the robustness of the phylogeny but only to test the consistence between different software.]<br><br>>>Response: Sorry for the missing information and we have added the your requested information in the revised manuscript. In brief, the initial orthologs-based tree was generated directly by fasttree built-in OrthoFinder. To illustrate the robustness of this topology based on orthologs genes, we reconstructed the coalescent and concatenate trees using IQtree and RaxML, respectively, with all bootstrap values 100. Also, we generated a large number of trees representing all possible topologies using densitree to exclude interference from gene flow. These results are all consistent, suggesting a reliable result based on the 180 nuclear genes.<br><br><br>[The authors included 10 lines to infer the global origin and dispersal of water hyacinth and concluded that "water hyacinth originates mainly from a single dispersal event from Brazil". Firstly, the sampling size is too small to derive such conclusion. Secondly, the chloroplast genomes are almost identical, with only a 1bp indel, which doesn't provide much information about phylo-geographic history. Thirdly, in the phylogeny (figure 4b), a Brazil line was put at the root. What's the justification? At least an outgroup is needed to root the tree. In addition, information about the chloroplast genome assembly, SNP calling, phylogeny inference etc. are missing.]<br><br>>>Response: We agree your point about sampling size and our conclusion about water hyacinth's dispersal. We are collecting more water hyacinth globally and plan to do a global population investigation by genome re-sequencing. The water hyacinth is an famous invasive plant, which happened about one hundred years ago from south

America and it will be interesting to observe genomic changes during the 100 years global dispersal. The 10 lines used in this study, we mainly used to estimate genetic diversity of water hyacinth. We have changed our statement about the conclusion of water hyacinth dispersal in the revised version.
We agree your point about the outgroup of phylogeny. As previously stated in our manuscript, the absence of genomic data on closely related Commelinales restricted us to use species beyond the genus for constructing a whole-genome sequence-based tree. Given that Brazil, located in South America, is widely acknowledged as the country of origin for water hyacinth, we opted to utilise a Brazilian species as the root taxon for our phylogenetic tree. This enables us to basically showcase the phylogenetic relationship between various lines. We have added the methods related to chloroplast genome assembly and other missing information in the revised version.

[Line 55: Can the authors explain a little bit about the taxonomy? Which name is accepted, which is synonym? If a formal taxonomic revision has been done, synonym name is not necessary here.]

>>Response: Thanks for your suggestions. Eichhornia crassipes (Mart.) Solms is as homotypic synonym of Pontederia crassipes Mart, which was revised in Taxonomy database of NCBI recently. Following your suggestion, we used Pontederia crassipes rather than Eichhornia crassipes in the revised version. However, the "Eichhornia crassipes" is the most commonly Latin name used in the current available scientific literature (several examples as followed). Regarding to consistency and clarity, we also provided the synonym in the context.

Monroy-Licht A, Méndez-Cuadro D, Olivero-Verbel J. Elemental mercury accumulation in Eichhornia crassipes (Mart.) Solms-Laubach. Environ Sci Pollut Res Int. 2023 Jan;30(4):9898-9913. doi: 10.1007/s11356-022-22521-y. Epub 2022 Sep 6. PMID: 36064851.
Feng W, Xiao K, Zhou W, Zhu D, Zhou Y, Yuan Y, Xiao N, Wan X, Hua Y, Zhao J. Analysis of utilization technologies for Eichhornia crassipes biomass harvested after restoration of wastewater. Bioresour Technol. 2017 Jan;223:287-295. doi: 10.1016/j.biortech.2016.10.047. Epub 2016 Oct 19. PMID: 27780621.

[Line 130: Phylogenetic position of E. crassipes?]

>>Response: Thank you for the above suggestions. We have modified the text accordingly.

[Line 131-137: This analysis with only eight species doesn't say much about the phylogeny of commelinids. To discuss the commelinids phylogeny, a proper sampling is needed, at least major clades in family level should be included.]

>>Response: Thanks for your suggestions. As mentioned in our manuscript, this is the first reference genome of the Commelinales and no genome of other families are available. We hope that the representative genome by this study will partially resolve the phylogenetic relationships of the Commelinales. Undoubtedly, we need more genomes of other families to fully resolve the phylogenetic relationships of the Commelinids in future.

[Line 131-137: The phylogenetic analysis is not clear. How did you construct the tree using 180 single-copy orthologs? Concatenated or coalescent? Information on alignment, model test, bootstrap etc. is missing.]

>>Response: Sorry for the confusion and we have added the missing information in the revised version. As mentioned above, this is the ultra-metric tree, transformed from the ML tree constructed based on concatenated sequence of 180 single-copy genes aligned by MAFFT. Bootstrap and model information about the ML tree have been provided in Supplementary Figure 3.
[Line 384: which database was used for BUSCO?]

>>Response: The database embryophyta_odb10 was used and we have added the information in the revised manuscript.

Powered by Editorial Manager® and ProduXion Manager® from Aries Systems Corporation

[Line 412: this is secondary calibration based on previous studies, rather than fossil constraint.]

>>Response: Thank you so much for your careful check. We have modified in the revised version.

[Figure 1A. Is this a concatenated or coalescent tree? Consensus or ML-best tree? Did you do bootstrap analysis on this?]

>>Response: Sorry for the inconvenience and we have added the information in the figure.

Reviewer #2:
[Line 43: I am not sure what is meant by "likely a result of fitness requirement for its role as a weed". What is a fitness requirement and a weedy habit have to do with each other? Please clarify.]

>>Response: Previous studies have revealed that the costs of resistance genes in plants. For example, the widely deployed mlo powdery mildew resistance gene in barley is associated with necrotic flecking and yield loss. Unlike crops, in which strong resistance is a necessary agronomic trait, weeds usually require rapid growth and massive reproduction. In this way, natural selection in weeds may favour reduced investment in resistance in order to maximize their reproductive capacity.

[Line 58: Please clarify if it is allo or auto polyploid here.]

>>Response: Sorry for the confusion. It's an allotetraploid.

[Line 68:it is unclear from how this is written if rice, wheat and maize are specifically in the Commelinales or the more general commelinids clade with how this is written. Please specify "this clade" on line 69.]

>>Response: Thank you for pointing out this problem in the manuscript. We have re-written this part in the revised manuscript.

[Line 88: In general, the results had a lot of data interpretation and what I would consider discussion/conclusions. I suggest either having a single results and discussion section or being careful in the results when data is being interpreted and hypotheses are being presented.]

>>Response: Thank for your comments. We have adjusted the discussion section based on your comments and have moved some data interpretation in Results section to the Discussion section.

[Line 236-237: Highly speculative hypothesis. I would not say "likely" without some support or evidence. "Possibly" would be better. Better yet, maybe make it clear this is a hypothesis that needs testing.]

>>Response: Thanks for your suggestions. We have modified them in the revised version.

[Line 320: Could it also be gene loss due to no selection pressure in the maintainence of these genes? Genes will pseudogenize if they do not neo or sub functionalize and there is no selection to maintain them. It could be that these disease genes have low rates of neofunctionalization for one reason or another compared with other gene families. I would be extremely wary of prescribing a negative fitness cost when simply the lack of a positive fitness explains the same phenomenon. Also the point you bring on line 329-330 is also very good. Why maintain that which has no function; it should disappear due to drift.]

>>Response: Thank you for your rigorous comment. I'm agree with your speculation about the reasons of genes loss. In fact, there are multiple possibilities that could lead to a reduction in weed disease resistance genes. Fitness costs were found in a wide

Powered by Editorial Manager® and ProduXion Manager® from Aries Systems Corporation

	range of weeds, so we hypothesize that this is one explanation for this phenomenon<br><br>[Line 324: Clarify you mean pathogen resistant and not herbicide resistant in canary grass as we are talking about weeds.]<br><br>>>Response: We have re-written this part according to your comments.
Additional Information:	
Question	Response
Are you submitting this manuscript to a special series or article collection?	No
**Experimental design and statistics**<br><br>Full details of the experimental design and statistical methods used should be given in the Methods section, as detailed in our Minimum Standards Reporting Checklist. Information essential to interpreting the data presented should be made available in the figure legends.<br><br>Have you included all the information requested in your manuscript?	Yes
**Resources**<br><br>A description of all resources used, including antibodies, cell lines, animals and software tools, with enough information to allow them to be uniquely identified, should be included in the Methods section. Authors are strongly encouraged to cite Research Resource Identifiers (RRIDs) for antibodies, model organisms and tools, where possible.<br><br>Have you included the information requested as detailed in our Minimum Standards Reporting Checklist?	Yes
**Availability of data and materials**<br><br>All datasets and code on which the conclusions of the paper rely must be either included in your submission or deposited in publicly available repositories (where available and ethically	Yes

appropriate), referencing such data using a unique identifier in the references and in the “Availability of Data and Materials” section of your manuscript.<br><br>Have you have met the above requirement as detailed in our Minimum Standards Reporting Checklist?	

Manuscript

Click here to access/download;Manuscript;Water hyacinth genome - Feb 8.docx

A reference genome of Commelinales provides insights into the commelinids evolution and global spread of water hyacinth (*Pontederia crassipes*)

Yujie Huang[1,2,10], Longbiao Guo[3,10], Lingjuan Xie[1], Nianmin Shang[1], Dongya Wu[1], Chuyu Ye[1], Eduardo Carlos Rudell[4], Kazunori Okada[5], Qian-Hao Zhu[6], Beng-Kah Song[7], Daguang Cai[8], Aldo Merotto Junior[4], Lianyang Bai[9,*], Longjiang Fan[1,2,*]

[1]Institute of Crop Sciences & Institute of Bioinformatics, Zhejiang University, Hangzhou 310058, China

[2]Zhongyuan Institute of Zhejiang University, Zhengzhou 450000, China

[3]State Key Laboratory of Rice Biology, China National Rice Research Institute, Hangzhou 310006, China

[4]Department of Crop Sciences, Agricultural School, Federal University of Rio Grande do Sul, Porto Alegre, RS. Brazil

[5]Agro-Biotechnology Research Center (AgTECH), University of Tokyo, Tokyo 113-8657, Japan

[6]CSIRO Agriculture and Food, Black Mountain Laboratories, Canberra, ACT 2601, Australia

[7]School of Science, Monash University Malaysia, 46150 Bandar Sunway, Selangor, Malaysia

[8]Department of Molecular Phytopathology and Biotechnology, Christian Albrechts University of Kiel, Kiel D-24118, Germany

[9]Hunan Weed Science Key Laboratory, Hunan Academy of Agriculture Science, Changsha, 410125, China

[10]Equal contributions

*Correspondence: fanlj@zju.edu.cn (L.Fan) and lybai@hunaas.cn (L.Bai)

Yujie Huang [0000-0002-7310-6178];

Longbiao Guo [0000-0003-1016-049X];
Lingjuan Xie [0009-0000-3594-6427];
Nianmin Shang [0009-0008-9838-6074];
Dongya Wu [0000-0003-1967-2264];
Chuyu Ye [0000-0003-0903-0356];
Eduardo Carlos Rudell [0000-0002-4296-2318];
Kazunori Okada [0000-0001-7321-7911];
Qian-hao Zhu [0000-0002-6505-7417];
Beng-Kah Song [0000-0001-9813-4557];
Daguang Cai [0000-0002-1816-6389];
Aldo Merotto Junior [0000-0002-1581-0669];
Lianyang Bai [0000-0002-4318-030X];
Longjiang Fan [0000-0003-4846-0500]

Abstract

Commelinales belongs to the commelinids clade which also comprises Poales that includes the most important monocot species, such as rice, wheat, and maize. No reference genome of Commelinales is current available. Water hyacinth (*Pontederia crassipes or Eichhornia crassipes*), a member of Commelinales, is one of the devastating aquatic weeds although it is also grown as an ornamental and medical plant. Here, we present a chromosome-scale reference genome of the tetraploid water hyacinth with a total length of 1.22 Gb (over 95% of the estimated size) across eight pseudochromosome pairs. With the representative genomes, we reconstructed phylogeny of the commelinids, which supported Zingiberales and Commelinales being sister lineages of Arecales and shed lights on the controversial relationship of the orders. We also reconstructed ancestral karyotypes of the commelinids clade and confirmed the ancient commelinids genome having eight chromosomes but not five as previously reported. Gene family analysis revealed contraction of disease-resistance genes during polyploidization of water hyacinth, likely a result of fitness requirement for its role as a weed. Genetic diversity analysis using nine water hyacinth lines from three continents (South America, Asia and Europe) revealed very closely related nuclear genomes and almost identical chloroplast genomes of the materials, and provided clues about the global dispersal of water hyacinth. The genomic resources of *P. crassipes* reported here contribute a crucial missing link of the commilinids species and offer novel insights into their phylogeny.

Keywords:

Pontederia crassipes or Eichhornia crassipes; Commelinales; reference genome; phylogeny of the commelinids; genetic diversity; karyotypes

Introduction

Pontederia crassipes (NCBI: txid44947, former name *Eichhornia crassipes*) , commonly known as water hyacinth, belongs to Pontederiaceae of the Commelinales, and is a perennial floating plant with light blue or purple flowers. *P. crassipes* is an allopolyploid with 32 chromosomes (2n = 4x = 32) [1]. Water hyacinth originated from Amazon Basin, South America and has spread to the tropics and subtropics since the 1800s to have a pan-tropical distribution across the world [2]. It is recognized as an exceedingly aggressive aquatic plant species that exhibits rapid growth and possesses the capacity for both sexual and asexual reproduction [3]. Though restricted to freshwater environments, it can effectively utilize nutrients so flourishes particularly in ecosystems with high nutrient loading, consequently outcompeting native plant species for space and sunlight [3–6]. As a result, it has been recognized by the International Union for Conservation of Nature as one of the 100 most invasive species and has been listed among the ten most serious weed plants in the world [7,8].

Commelinales is a branch of the commelinids clade, which also comprises Poales, Zingiberales and Arecales. Many members of commelinids, such as rice, wheat and maize, provide calorie-rich grains, livestock feed, and industrial raw materials [9–11]. A phylogenic tree of the commelinids has been constructed based on plastid genomes [12]. However, the controversy surrounding the phylogeny of commelinids, especially the placement of Poales and Commelinales, persists [12–14]. This uncertainty is attributed to discordance between nuclear and organellar phylogenies, which may arise from hybridization, incomplete lineage sorting, gene duplication, and gene loss [15–17]. Nuclear–plastid conflicts are prevalent at different taxonomic levels of angiosperms, such as the placement of COM (Celastrales, Malpighiales, and Oxalidales) clade and the commelinids [13,18,19]. Many genomes of the economically important members of the commelinids have been sequenced, for example the grasses (Poaceae), gingers and bananas (Zingiberales), and palms (Arecaceae). However, no reference genome within the Commelinales order has been generated up to now, which has

hindered the elucidation of the phylogenetic puzzle of commelinids.

Here, we generated a chromosome-scale genome assembly of *P. crassipes*, investigated genome evolution of *P. crassipes* in relation to its related species to determine the phylogeny and ancestral karyotype of the commelinids, and further explored the genetic diversity and phylogenetic relationships of water hyacinth using materials collected from several countries.

Results

Genome assembly, phasing and annotation

We sequenced an *P. crassipes* individual (Zijingang#1) collected from Hangzhou, China. The estimated genome size of *P. crassipes* was ~1,058 Mb based on *k*-mer survey using Illumina short reads (Figure S1A), and ~1,278 Mb based on flow cytometry, consistent with its C-value of 1.28 pg/1C [20] (Figure S1B-D). The heterozygosity level of the *P. crassipes* genome was estimated to be 0.76% and repetitive content accounts for 68.85% of the genome (Figure S1A). Based on 68 Gb (52×) HiFi reads with an average read length of 17.72 kb, a *de novo* assembly yielded a genome of 1.30 Gb, including 1,699 contigs with a contig N50 size of 39.5 Mb (Supplementary Table S1).

With the 130 Gb Hi-C data generated by this study, we assembled the genome of *P. crassipes* by anchoring 606 contigs to 16 superscaffolds (pseudochromosomes) with a total length of 1.22 Gb, representing 95.3% of the estimated size (Table 1). Attributed to the nature of tetraploid, the high collinearity between the two subgenomes brings challenges to assembly, which severely reduced the reliability of the regular ordering methods. Given that allopolyploids containing subgenome-specific sequences, we searched subgenome-specific sequence (*k*-mer) and then clustered the specific sequences that differentiate homoeologous chromosomes, which enabled consistent partitioning of the genome into two subgenomes (Figure S2). Consequently, 16 superscaffolds were assigned to the two subgenomes, termed as subA and subB. After phasing and ordering with directional interactions, we finally assembled the genome of

P. crassipes with the size of subA and subB being 640.2 Mb and 577.6 Mb, respectively, and the size of pseudochromosomes ranged from 45.81 to 104.49 Mb (Table 1).

The quality of the assembly was validated through mapping 98.51% of the genomic short reads obtained by Illumina sequencing to the assembly. The long terminal repeat (LTR) assembly index (LAI) score was 11.78, indicating a reference quality, comparable with those of Arabidopsis (TAIR10) and *Vitis vinifera* [21,22]. We also estimated base-level accuracy and completeness of the genome and achieved a high assembly consensus quality value (QV=42.0). High genomic synteny was observed between the *P. crassipes* assembly and other genomes within the commelinids clade (e.g., *Cocos nucifera*) (further details are available in the following sections). Taken together, the results suggested the reliability of the *P. crassipes* assembly.

A total of 65,299 genes were predicted in the *P. crassipes* assembly by applying a combination of homology, transcript-based and *ab initio* gene predictions approaches, after filtering out 732.02 Mb (56.09%) of repetitive sequences. Subsequently, we identified 33,608 and 31,691 genes in subgenomes A and B, respectively. Benchmarking Universal Single-Copy Orthologue (BUSCO) was used to assess the completeness of our genome annotation, which revealed that the gene set we annotated encompassed 1,536 (95.2%) of the 1,614 universal single-copy genes present in the Embryophyta lineage [23] (Supplementary Table S2).

Phylogenetic position of *P. crassipes* based on single-copy genes

To resolve the phylogenetic position of Commelinales in the commelinids, we firstly constructed a phylogenetic tree using concatenated sequence of 180 single-copy orthologs identified by OrthoFinder [24] of the water hyacinth genome and other seven representative members with high-quality genomes, using *Acorus tatarinowii* as the outgroup (Figure S3A). The phylogenetic tree revealed that Zingiberales and Commelinales were sister lineages of Arecales, and Poales is located in the out node, which supports the previous phylogenetic studies by Cheng et al and Wang et al [25,26]. To validate the stability of the phylogenetic tree, we reconstructed a maximum-

likelihood (ML) phylogeny by utilizing a concatenated matrix comprising 180 single-copy orthologs from the nine genomes. A coalescent-based phylogeny was also generated through integration of the single-copy gene trees (Figure S3B, C). The topologies of both the coalescent and concatenate trees supported the aforementioned orthologs-based tree. Strong robustness was evident at each node (Figure S3B) within the coalescent tree. At the same time, the outcomes were also in accordance with a consensus tree generated using DensiTree [27] (Figure S3D).

To date evolutionary events, we reconstructed a time-calibrated phylogenetic tree combined with fossil calibration time (Figure 1A). The analysis showed an early origin of commelinids in Jurassic, ~160 million years ago (mya) (136.6 – 201.3 under 95% CI) (Figure 1A). And Commelinales arose at ~87.7 mya (82.1 – 93.2) and the divergence time between subgenome A and B of *P. crassipes* was dated at ~6.4 mya (4.4 – 9.0).

Whole genome duplications of *P. crassipes*

Whole genome duplications (WGDs) cause rapid genome reorganization and structural variations to produce new chromosomal karyotypes [28,29]. The analysis of genomic synteny showed excellent collinearity within the *P. crassipes* genome, which suggested recent genomic duplication events (Figure S4). Based on the syntenic blocks, we clustered the pseudochromosomes into ancestral chromosomes as A1 (Chr1A ~ 4A), A2 (Chr5A ~ 8A), B1 (Chr1B ~ 4B) and B2 (Chr5B ~ 8B). To confirm potential WGDs events in the water hyacinth genome and estimate divergence time, we extracted syntenic gene pairs within the *P. crassipes* genome and their orthologs in four representative species of the commelinids (*A. tatarinowii*, *Musa balbisiana*, *C. nucifera* and *Pharus latifolius*). The distribution of synonymous substitutions per site (K_S) indicated that at least three rounds of WGDs happened during *P. crassipes* evolution, consistent with the above synteny analysis results (Figure 1B). However, the estimated divergence of water hyacinth and palms of Arecaceae (K_S = 1.04) occurred after divergence from Zingiberales (K_S = 1.17) according to the K_S peaks, which conflicts

with the phylogenetic tree (Figure 1A). The stronger collinearity between water hyacinth and palms seemed to support the result of K_S distribution (Figure 1C).

The conflict between the K_S inference and phylogenetic analysis might be triggered by several factors, such as different substitution rates or structural genomic rearrangement rates [30,31]. To test this hypothesis, we inferred the substitution rate in each branch with Bayesian methods implemented in BEAST2 [32]. Concordant with the hypothesis, the estimated substitution rate in the palm (0.67) was significantly less than that in the ginger (1.18), indicating that the evolutionary rate variation across the taxa caused the bias of the K_S distribution.

We further extracted paralogs present in the genomes derived from the WGDs, aiming to elucidate the orders and dates of the WGD events that transpired during the evolution of water hyacinth. Two prominent peaks of the K_S distribution of water hyacinth (Figure 1B) suggested two relatively recent WGD events. These events encompassed the most recent tetraploidization event and a duplication event specific to the Commelinales lineage. Water hyacinth shared an ancient WGD with other commelinids, which has been recognized as the τ WGD event [26] (Figure S5). To confirm the ancient duplication process, we estimated the copy number in collinear regions between the water hyacinth and coconut genomes and found that some genomic regions indeed shared four corresponding copies in the two genomes (Figure 1C). A case with the detailed genomic synteny between the two genomes (water hyacinth Chr3, Chr4, Chr6, Chr7 vs coconuts Chr4, Chr12, Chr16) is shown in Figure 1C. Following the estimated time of *τ* WGD (129-146 mya) based on the coconut genome [26], the tetraploidization event of water hyacinth was estimated to occur approximately 8-10 mya and the lineage-specific duplication at 67-76 mya, which all were comparable to the phylogenetic estimates (Figure 1A). Differentiated transposable element (TE) contents were observed in two subgenomes of water hyacinth, with a divergence rate ranging from 2% to 8% (subA) and 16% to 22% (subB). These differences resulted in the formation of a distinctive “bubble” peak within the TE profile, indicating a WGD pattern similar to that observed in the analysis of colinear paralogous pairs (Figure S6).

Mass loss of disease-resistance genes in the *P. crassipes* genome

To estimate gene loss and gain during polyploidization, gene family sizes were determined by identifying protein domains in *P. crassipes* and other representative genomes. We first compared gene family sizes between tetraploid *P. crassipes* and the diploid *Oryza sativa* genome using a dot matrix plot (Figure 2A). The results showed that the size of the majority of gene families in *P. crassipes* was almost two times higher than those in *O. sativa*, consistent with their ploidy. The analysis also revealed that the size of several gene families (predominantly associated with disease-resistance) in *P. crassipes* was significantly smaller than expected, e.g., genes encoding NB-ARC (226 in *P. crassipes* versus 522 and 480 in *O. sativa* and another diploid grass *Setaria italica*, respectively), GRAS (62 versus 65 and 59, respectively), peroxidase (162 versus 158 and 170, respectively) and legume lectin (66 versus 99 and 63, respectively) (Figure 2E) [33–37]. We also compared the gene family size between water hyacinth and two other species of the grass family, tetraploid weed *Echinochloa oryzicola* (Figure 2A) and crop durum wheat (*Triticum turgidum*), and found the same trend (Figure 2B). For example, the number of NB-ARC genes in durum wheat (753) and *E. oryzicola* (318) was higher than in water hyacinth (226) ($P < 0.001$, Fisher's exact test). The results suggested a contraction of disease-resistance genes in the *P. crassipes* genome, consistent with the phenomenon observed in the *Echinochloa* weeds [38].

To estimate the loss/gain of disease-resistance genes during duplication, we calculated synteny retention ratios of collinear gene pairs in the water hyacinth genome by estimating the percentage of the retained gene pairs experienced the two polyploidization events (A1:A2:B1:B2 = 1:1:1:1) and one of the two events (A1:A2 or B1:B2 = 1:1) as well as between the two subgenomes (subA:subB = 1:1) (Figure 2C-E). Across the genome, while 23.2% of genes fit the 1:1:1:1 (A1:A2:B1:B2) synteny retention ratio (Figure 2C), the synteny retention ratio of the NB-ARC family genes (3.8%) was significantly lower ($P < 0.0001$, Fisher's exact test); similarly, a significantly low synteny retention ratio (3.1%; $P < 0.0001$) was also evident for

another well-known disease-resistance gene family, the wall-associated receptor kinases [39]. To identify the conversation pattern of the gene families after polyploidization events, we also compared synteny retention ratios of collinear gene pairs originated from different events (i.e., A1:A2 or B1:B2 = 1:1). The results illustrated that genes encoding NB-ARC (7%, $P < 0.0001$; 44%, $P < 0.01$) and wall-associated receptor kinases (10.1%, $P < 0.0001$; 47%, $P < 0.01$) suffered significant loss after polyploidization (Figure 2D, E).

The same bioinformatics pipeline was used to compare the patterns of gene retention and loss in the commelinids for several other gene families. A higher number of P450 genes (574) was observed in *P. crassipes* compared with other species, possibly related to its capacity of survival in the severely polluted conditions (Figure 2B). In Arecales and Zingiberales, the increased number of GRAS genes implying a reduction of the gene family during divergence of *P. crassipes* (Figure 2B). Consistent with the findings from previous studies [40–42], we observed a significant increase of disease-resistance genes in crops, including genes encoding legume lectin, peroxidase, and NB-ARC.

Ancestral karyotype evolution of the commelinids

Being a key phylogenetic branch within the commelinids clade, the high-quality reference genome of Commelinales generated in this study provides an opportunity to reconstruct the ancestral karyotype of the commelinids. We therefore compared seven representative species with well-assembled genomes with *P. crassipes* (Figure 3A). By inferring intergenomic gene collinearity, we mapped the seven genomes onto *P. crassipes*, and estimated the ratio of the best-matched orthologous regions between *P. crassipes* and *C. nucifera* (Arecaceae), *A. comosus* (Poaceae), and *Z. officinale* (Zingiberales) being 4:2, 4:3 and 4:4, respectively, a result consistent with the WGD times experienced by the species (Figure S7A). Based on the gene collinearity of the four genomes (Figure 3A, Figure S13a-c), we constructed an ancestral karyotype with 8 proto-chromosomes shared by the commelinids (Figure 3B). Accordingly, we also reconstructed the ancestral karyotypes of other four species, *O. sativa*, *M. balbisiana*,

Brachypodium distachyon and *P. latifolius*.

The reconstruction results clearly showed frequent chromosomal rearrangements in *P. crassipes* and genome structure changes in Zingiberales (Figure 3B). A close check of shared collinearity between extant plant chromosomes identified the origin of certain extant chromosomes thereby revealing their antiquity. For example, the region originated from τ WGD located in the chromosome 6 of *C. nucifera* and chromosome 1 of *P. crassipes* (Figure 3A). From the deduced ancestral state, Commelinales proto-chromosomes have been shaped through *τ* WGD followed by 1 fission and 13 fusions to reach an $n = 4$ intermediate state. Then 3 fissions and 13 fusions accounted for the transition between the $n = 4$ intermediate state and the modern genome structure of 8 chromosomes in subgenome A and B of *P. crassipes*. The fewest chromosomal rearrangements were observed in *C. nucifera*, consistent with its low nucleotide substitution rate, while Zingiberales underwent similar massive chromosomal rearrangements.

Genetic diversity of *P. crassipes*

To estimate genetic diversity of global water hyacinth, we collected additional nine lines from South America (Brazil), Asia (China and Malaysia) and Europe (Germany) (Figure 4) and sequenced them with an average of 36× genomic coverage. Based on the SNPs amongst the nine genomes and the *P. crassipes* reference genome (Zijingang#1), we found a relatively low genetic diversity (π = 1.44×10^{-3}) of the global water hyacinth, comparing to sorghum (3.05×10^{-3}) and other crops [43]. Based on principle component analysis (PCA), the water hyacinth native to Brazil (five lines from different locations) seemed to have a relatively higher diversity than those from other countries (Malaysia, China and Germany) (Figure S8), indicating a tendency of a more divergent genetic diversity of the species in the area of its origin [44].

The phylogenetic tree of the global water hyacinth was consistent with the PCA results (Figure 4), in which the Brazil lines embraced the three lines from other three countries. Of all five non-Brazilian lines, except one of the lines from Germany

(Germany_Rostock), the other four lines (including the Zijingang#1 line) had an almost same nuclear genome to two Brazilian lines (Brazil_Vicosa and Brazil_Bombinhas). The chloroplast genomes of all ten lines were further assembled and, surprisingly, only two chloroplast genomes (named as chloroplast genome A and B) of water hyacinth which were nearly identical and differed by only a 1bp indel (Figure 4A), were achieved. In Brazil, the chloroplast genome A and B were observed in lines from the southern and northern areas, respectively, while all water hyacinth lines from other countries had the genome A. Taken together, these results support Brazil as one of origins of water hyacinth, and suggest a global spread potentially by one or two genotypes.

Discussion

At present, all of the major commelinids crops (e.g., rice, wheat and maize) [45–47] and other important economic crops of the clade such as pineapple and bananas [45,48,49] have had their genomes sequenced. However, the Commelinales order, an important phylogenetic node of the commelinids, still lacks a reference genome until now. Here we generated a high-quality reference genome of *P. crassipes*, representing the first genome of the Commelinales order. The availability of the genome provides a crucial missing link among different orders of the commilinids clade and is anticipated to facilitate studies of genome evolution.

The analysis on ancient karyotype of the commelinids provides clear evidence for the clade having eight proto-chromosomes. While the result differs from the result of five proto-chromosomes reported by other studies [50,51], it is in line with the result based on study of coconuts [26]. Apparently, the lack of high quality genomes of representative species of crucial nodes of phylogenetic tree hinders the inference of evolutionary framework [52]. With the continuously increasing number of high-quality genomes, particularly the genomes filling the missing links, such as the water hyacinth genome generated in this study, gene collinearity and syntenic blocks between different species of the commelinids clade can be more clearly defined and characterized, so to shed lights on the plasticity of the commelinids genomes and their evolutionary

trajectories.

Water hyacinth seemed to have experienced significant reduction in disease-resistance genes (such as NB-ARC, GRAS, peroxidase and legume lectin) during its evolutionary history. This could potentially be linked to fitness costs associated with allocating energy towards growth and reproduction processes [53–55]. Emerging data demonstrate that the growth-defense trade-offs allow plants to adjust growth and defense based on external conditions [56]. The phenomenon of shrinking of disease-resistance genes has also been observed in other noxious weeds [38,53–55].. It is reasonable to assume, therefore, that the loss of the disease-resistance gene in the *P. crassipes* genome could be a result of natural selection to maximize and accelerate the growth and reproduction of *P. Crassipes*. However, it is also possible that fewer disease-resistance genes evolved during its evolutionary history due to lower disease pressure in the surrounding environment (water) where *P. crassipes* grows. Significant contractions in certain disease-resistance gene families imply stronger competitiveness and invasiveness of *P. crassipes*. While strong disease-resistance is an important agronomic trait for crops, rapid growth and extensive reproduction may be necessary for weediness and invasiveness in general. Further investigation of the underlying mechanisms, such as fitness costs in weeds, will thus contribute to a better understanding of their invasive strategy and could potentially be used to develop effective weed management strategies.

This study revealed both identical nuclear and chloroplast genome between some of the Brazilian water hyacinth and all the water hyacinth from other countries (except the German Rostock line), indicating the spread of limited genotype of water hyacinth from South America, where has the highest genetic diversity. The genetic uniformity has been observed in global spread of water hyacinth and other invasive species [7,57]. Bombinhas is a city in the southern region of Brazil, located in close proximity to the Itajaí Port, the sixth largest port in Brazil, established in the early 1860s. Given the strategic location of the Itajaí Port on the South American East coast, there is a possibility that the early invasion abroad of water hyacinth could have facilitated by the

transportation/immigration from the Itajaí Port, which was not mentioned in Brazil history. Although the Rostock line may indicate additional global dispersal of water hyacinth, our results indicated that the available non-Brazilian water hyacinth may originated from Brazil

Materials and methods

Materials collection and sequencing

A wild *P. crassipes* plant (Zijingang#1) collected from Zijingang Campus of Zhejiang University, Hangzhou, China was used in construction of the reference genome. The additional nine lines of *P. crassipes* were collected globally for phylognetic analysis, with their detailed information available in Supplementary Table S3. Genomic DNA of *P. crassipes* was extracted from young leaves using the CTAB method for sequencing library construction. Following the standard protocols of the Pacific Biosciences Company, DNA libraries for single-molecule real-time (SMRT) PacBio genome sequencing were constructed and circular consensus sequencing (CCS) was performed on PacBio Sequel2 platform (RRID:SCR_017990) for high fidelity (HiFi) reads. Short-read libraries of *P. crassipes* were constructed according to Illumina's standard protocol, and paired-end reads (2 × 150 bp) were sequenced on an Illumina HiSeq X Ten platform (RRID:SCR_020131). With default parameters, raw PacBio subreads were filtered and corrected using the pbccs pipeline.

A Hi-C library was constructed using fresh young leaves of *P. crassipes*, which were fixed in 1% formaldehyde for crosslinking. Cells were lysed using a Dounce homogenizer and digested using the *Hin*d III restriction enzyme. The DNA ends were filled and labeled with biotin and the filled-in *Hin*d III sites were ligated to form *Nhe* I sites. Complexes with the biotin-labeled ligation products were purified and sheared, and the biotinylated Hi-C ligation products were pulled down and used to construct Illumina sequencing libraries [58].

Genome assembly

The HiFi reads were subjected to hifiasm (RRID:SCR_021069) [59] for *de novo*

assembly in default mode. After mapping the long subreads to the initial assembly with minimap2 (RRID:SCR_018550)[60], racon [61] was used in three rounds of correction with default parameters. Based on the subassembly, clean Hi-C reads were analyzed and 3D-DNA [62] was used to scaffold contigs into pseudochromosomes followed by manually corrected with Juicer (RRID:SCR_017226) [63].

The above genome assembly was subjected to SubPhaser [64] to search the subgenome-specific sequence (*k*-mer), and then homoeologous chromosomes were assigned into two subgenomes (Figure S2). Based on the coverage depth of the short reads against the assembly, we manually corrected some errors with discrete chromatin interaction patterns. The assembled genome was subjected to BUSCO v5.5.0 (RRID:SCR_015008) [23] with embryophyta_odb 10 to evaluate the completeness of the genome.

Genome annotation

Repeat families were first identified *de novo* and classified initially using RepeatModeler v1.0.10 (RRID:SCR_015027) [65]. The repeat library by RepeatModeler was analyzed with RepeatMasker v4.0.7 (RRID:SCR_012954) [65] for the whole genome repeat annotation.

A hybrid strategy integrating *ab initio* predictions by Fgenesh [66] and AUGUSTUS v3.2.2 (RRID:SCR_017555) [67], homologue evidence-based prediction, and transcript-assisted predictions was applied for gene prediction. EVidenceModeler v1.1.1 (RRID:SCR_014659) [68] was used to integrate the gene models predicted by the above approaches to obtain a non-redundant consensus gene set. Gene models were identified as those supported by homologous genes or transcript evidence or by at least two *ab initio* methods. High-confidence gene models were further filtered to remove short gene models (less than 50 amino acids) and gene models with homology to sequences in the Repbase (RRID:SCR_021169) (E value ≤ 1 × 10−5, identity ≥30%, coverage ≥25%). Functional annotations of protein-coding genes were conducted based on Pfam protein domains using InterProScan v5.24–63.0 (RRID:SCR_005829) [69].

Tandem repeats were identified with Satellite Repeat Finder (SRF) [70], and one type of centromere sequences was found. To precisely annotate the location of the

centromeric monomers *CEN148*, we calculated peak values in the windows of divided genome and merge the windows with the same kind of monomers.

Divergence time estimation

Phylogenetic trees for *P. crassipes* and seven other species (*M. balbisiana* [71], *Z. officinale* [25], *C. simplicifolius* [72], *C. nucifera* [26], *P. latifolius* [73], *O. sativa* [74] and *A. tatarinowii* [51]) were built with = FastTree [75] using 180 shared single-copy genes identified by OrthoFinder [24] and visualized in iTOL (itol.embl.de) [76]. The phylogenetic relationship was further checked by IQ-TREE 2 (RRID:SCR_017254) [77] with concatenated- and coalescent-based input data. The substitution rate in different branch was inferred in BEAST2 (RRID:SCR_017307)[32]. The species tree rooted with *A. tatarinowii* was used as an input to build an ultrametric tree by the MCMCTree program in PAML (RRID:SCR_014932) [78], whereas secondary calibration were set to *A. tatarinowii–O. sativa* (133.0 - 139.1 mya) derived from the Timetree database [79]. TE divergence was assessed by PercDivs (Percentage of substitutions in the matching region compared with the consensus) calculated in RepeatMasker. TE sequence divergence between two subgenomes of tetraploid *P. crassipes* displaying a high degree of overlap suggesting the consistency of the TE evolutionary rate in the two subgenomes (Figure S6). The non-overlapping segregation region represents the period between the divergence of diploid progenitors and the merging of their genomes into a tetraploid genome [80].

Genome polyploidization analysis

We selected four representative species including *M. balbisiana, C. simplicifolius, P. latifolius*, and *A. tatarinowii*, for comparative genomics analysis with *E. crassipes*, aiming to investigate the polyploidization event(s) that occurred and whether they were shared or not, and to infer the evolutionary trajectories that led to the formation of current chromosomes. We first aligned protein sequences manually among species or subgenomes. WGDI was used to identify colinear blocks, which are the genomic regions containing colinear genes according to the combined information of gene similarity and gene order, within and between each genome [81]. The maximum gap

allowed between collinear genes on a chromosome was set to 50 intervening or non-colinear genes. To help date evolutionary events and identify colinear genes produced by different events, polyploidization or speciation, K_S between colinear genes were estimated using KaKs_calculator with the NG model [82]. Given that the possible effects of diverse nucleotide substitution among different lineages for phylogeny estimation, shared polyploidization between water hyacinth and coconut was recognized as an anchor to date duplication events occurred in water hyacinth.

Analysis of ancestral karyotypes and chromosome evolutionary trajectories

To investigate the chromosome evolution of commelinids genomes, we selected representative species (Figure 3B) from four orders with chromosome-level genome assembly. We identified homologous proteins between extant genomes and the reconstructed commelinids karyotypes, and then used WGDI to detect syntenic blocks as described above [81]. Then, dot plots were created to show synteny and the chromosomal rearrangements were reconstructed.

Gene family identification

InterProScan (version 5.24–63.0) [69] was used to identify Pfam protein domains, which were used to identify gene families. Besides the *P. crassipes* genes annotated in this study, protein domains were also identified for the genes of *P. latifolius* [73], *S. italica* (v2.0) [83], *O. sativa* [74], *T. turgidum* [46], *E. oryzicola* [84], *M. balbisiana* [71], *Z. officinale* [25], *Phoenix dactylifera* [85] and *C. nucifera* [26].

Resequencing and variant calls

For resequencing, short-read libraries of additional lines of *P. crassipes* collected globally were sequenced on an Illumina HiSeq X Ten platform (Supplementary Table S3). Raw data were first filtered by NGSQC Toolkit (v2.3.348)[86]. Clean paired-end reads of each accession were then aligned to *P. crassipes* using Bowtie2 with default parameters. A custom pipeline [87] was used in calling and filtering variants. Low-quality variants were further removed with minor allele frequency (MAF) < 0.01 and missing rate > 30%.

Phylogenetic analysis and PCA

The phylogenetic analysis was performed on the full set of all 9 water hyacinth lines. Phylogeny tree was constructed based on 6.5 million SNPs using FastTree (RRID:SCR_015501) [75] and visualized in iTOL (RRID:SCR_018174) [76]. All SNPs with lines were analyzed using the R package SNPRelate to conduct principal component analysis (PCA) [88].

Chloroplast genome assembly and annotation

The clean data of illumina sequencing reads of all nine lines was applied in *de novo* assembly by GetOrganelle (RRID:SCR_022963) [89]. Genome annotation was performed by the GeSeq (RRID:SCR_017336) online [90]. A custom script was used to filter out duplicate annotated genes. Multiple sequence alignment of chloroplast genomes was performed with MAFFT (RRID:SCR_011811) [91].

Data Availability

The genomic sequence and RNA-seq data of *P. crassipes* generated by this study were deposited into the NGDC database under the accession number PRJCA020146 and European Nucleotide Archive (ENA) BioProject: PRJNA1062020. The assembled chloroplast genome sequences and annotation information have been submitted in NGDC under accession numbers C_AA041877.1, C_AA041878.1 and C_AA041879.1. All additional supporting data are available in the *GigaScience* repository, GigaDB [92].

Acknowledgement

This study is partially supported by National Key Research and Development Program (SQ2022YFD1400042) and CIC-MCP. We thank Susanne Petersen (Botanic Institute and Botanic Garden, Kiel University) and Malaysian Agricultural Research & Development Institute (MARDI) for their help in water hyacinth collection.

Reference

[1] Isa H, Egbuche KC, Malgwi MM, Tukur NA. Cytological studies in *Eichhornia crassipes*

(Mart.) Solms. Am J Plant Physiol 2013;8:50–62.

[2] Gopal B. Water hyacinth. Amsterdam ; New York : Elsevier ; New York, N.Y., U.S.A. : Distributors for the United States and Canada : Elsevier Science Pub. Co.; 1987.

[3] Villamagna AM, Murphy BR. Ecological and socio-economic impacts of invasive water hyacinth (*Eichhornia crassipes*): a review. Freshw Biol 2010;55:282–98.

[4] Cilliers CJ. Biological control of water hyacinth, *Eichhornia crassipes* (Pontederiaceae), in South Africa. Agric Ecosyst Environ 1991;37:207–17.

[5] Heard TA, Winterton SL. Interactions between nutrient status and weevil herbivory in the biological control of water hyacinth. J Appl Ecol 2000;37:117–27.

[6] Xie Y, Wen M, Yu D, Li Y. Growth and resource allocation of water hyacinth as affected by gradually increasing nutrient concentrations. Aquat Bot 2004;79:257–66.

[7] Zhang Y-Y, Zhang D-Y, Barrett SCH. Genetic uniformity characterizes the invasive spread of water hyacinth (*Eichhornia crassipes*), a clonal aquatic plant. Mol Ecol 2010;19:1774–86.

[8] Patel S. Threats, management and envisaged utilizations of aquatic weed *Eichhornia crassipes*: an overview. Rev Environ Sci Biotechnol 2012;11:249–59.

[9] Semwal RB, Semwal DK, Combrinck S, Viljoen AM. Gingerols and shogaols: Important nutraceutical principles from ginger. Phytochemistry 2015;117:554–68.

[10] Rahman H, Vikram P, Hammami Z, Singh RK. Recent advances in date palm genomics: A comprehensive review. Front Genet 2022;13.

[11] Kellogg EA. Evolutionary history of the grasses. Plant Physiol 2001;125:1198–205.

[12] Ma Q, Lu Y. The complete chloroplast genome of *Eichhornia crassipes*

(Pontederiaceae) and phylogeny of commelinids. Mitochondrial DNA Part B 2019;4:3186–7.

[13] Group TAP. An update of the Angiosperm Phylogeny Group classification for the orders and families of flowering plants: APG IV. Bot J Linn Soc 2016;181:1–20.

[14] Luo Y, Lu L, Wortley AH, Li D-Z, Wang H, Blackmore S. Evolution of angiosperm pollen. 3. monocots. Ann Mo Bot Gard 2015;101:406–55.

[15] Galtier N, Daubin V. Dealing with incongruence in phylogenomic analyses. Philos Trans R Soc B Biol Sci 2008;363:4023–9.

[16] Soltis PS, Soltis DE. The role of hybridization in plant speciation. Annu Rev Plant Biol 2009;60:561–88.

[17] Smith SA, Moore MJ, Brown JW, Yang Y. Analysis of phylogenomic datasets reveals conflict, concordance, and gene duplications with examples from animals and plants. BMC Evol Biol 2015;15:150.

[18] Guo C, Luo Y, Gao L-M, Yi T-S, Li H-T, Yang J-B, et al. Phylogenomics and the flowering plant tree of life. J Integr Plant Biol 2023;65:299–323.

[19] Li H-L, Wu L, Dong Z, Jiang Y, Jiang S, Xing H, et al. Haplotype-resolved genome of diploid ginger (*Zingiber officinale*) and its unique gingerol biosynthetic pathway. Hortic Res 2021;8:1–13.

[20] Pellicer J, Leitch IJ. The Plant DNA C-values database (release 7.1): an updated online repository of plant genome size data for comparative studies. New Phytol 2020;226:301–5.

[21] Jaillon O, Aury J-M, Noel B, Policriti A, Clepet C, Casagrande A, et al. The grapevine

genome sequence suggests ancestral hexaploidization in major angiosperm phyla. Nature 2007;449:463–7.

[22] Lamesch P, Berardini TZ, Li D, Swarbreck D, Wilks C, Sasidharan R, et al. The Arabidopsis Information Resource (TAIR): improved gene annotation and new tools. Nucleic Acids Res 2012;40:D1202–10.

[23] Manni M, Berkeley MR, Seppey M, Simão FA, Zdobnov EM. BUSCO Update: Novel and Streamlined Workflows along with Broader and Deeper Phylogenetic Coverage for Scoring of Eukaryotic, Prokaryotic, and Viral Genomes. Mol Biol Evol 2021;38:4647–54.

[24] Emms DM, Kelly S. OrthoFinder: solving fundamental biases in whole genome comparisons dramatically improves orthogroup inference accuracy. Genome Biol 2015;16.

[25] Cheng S-P, Jia K-H, Liu H, Zhang R-G, Li Z-C, Zhou S-S, et al. Haplotype-resolved genome assembly and allele-specific gene expression in cultivated ginger. Hortic Res 2021;8:1–15.

[26] Wang S, Xiao Y, Zhou Z-W, Yuan J, Guo H, Yang Z, et al. High-quality reference genome sequences of two coconut cultivars provide insights into evolution of monocot chromosomes and differentiation of fiber content and plant height. Genome Biol 2021;22:304.

[27] Bouckaert RR. DensiTree: making sense of sets of phylogenetic trees. Bioinformatics 2010;26:1372–3.

[28] Qiao X, Li Q, Yin H, Qi K, Li L, Wang R, et al. Gene duplication and evolution in recurring polyploidization–diploidization cycles in plants. Genome Biol 2019;20:38.

[29] Qiao X, Zhang S, Paterson AH. Pervasive genome duplications across the plant tree

of life and their links to major evolutionary innovations and transitions. Comput Struct Biotechnol J 2022;20:3248–56.

[30] Park D, Jung JW, Choi B-S, Jayakodi M, Lee J, Lim J, et al. Uncovering the novel characteristics of Asian honey bee, *Apis cerana*, by whole genome sequencing. BMC Genomics 2015;16:1.

[31] Lanfear R, Ho SYW, Jonathan Davies T, Moles AT, Aarssen L, Swenson NG, et al. Taller plants have lower rates of molecular evolution. Nat Commun 2013;4:1879.

[32] Bouckaert R, Vaughan TG, Barido-Sottani J, Duchêne S, Fourment M, Gavryushkina A, et al. BEAST 2.5: An advanced software platform for Bayesian evolutionary analysis. PLOS Comput Biol 2019;15:e1006650.

[33] Asada K. Ascorbate peroxidase – a hydrogen peroxide-scavenging enzyme in plants. Physiol Plant 1992;85:235–41.

[34] Werck-Reichhart D, Feyereisen R. Cytochromes P450: a success story. Genome Biol 2000;1:reviews3003.1.

[35] Meyers BC, Kaushik S, Nandety RS. Evolving disease resistance genes. Curr Opin Plant Biol 2005;8:129–34.

[36] Lannoo N, Van Damme EJM. Lectin domains at the frontiers of plant defense. Front Plant Sci 2014;5.

[37] Yu Q, Powles S. Metabolism-Based herbicide resistance and cross-resistance in crop weeds: A threat to herbicide sustainability and global crop production. Plant Physiol 2014;166:1106–18.

[38] Ye C-Y, Wu D, Mao L, Jia L, Qiu J, Lao S, et al. The genomes of the allohexaploid

Echinochloa crus-galli and its progenitors provide insights into polyploidization-driven adaptation. Mol Plant 2020;13:1298–310.

[39] Hurni S, Scheuermann D, Krattinger SG, Kessel B, Wicker T, Herren G, et al. The maize disease resistance gene *Htn1* against northern corn leaf blight encodes a wall-associated receptor-like kinase. Proc Natl Acad Sci 2015;112:8780–5.

[40] Van Der Biezen EA, Jones JDG. The NB-ARC domain: a novel signalling motif shared by plant resistance gene products and regulators of cell death in animals. Curr Biol 1998;8:R226–8.

[41] Hiraga S, Sasaki K, Ito H, Ohashi Y, Matsui H. A large family of class III plant peroxidases. Plant Cell Physiol 2001;42:462–8.

[42]Roopashree S, Singh SA, Gowda LR, Rao AGA. Dual-function protein in plant defence: seed lectin from *Dolichos biflorus* (horse gram) exhibits lipoxygenase activity. Biochem J 2006;395:629–39.

[43] Mace ES, Tai S, Gilding EK, Li Y, Prentis PJ, Bian L, et al. Whole-genome sequencing reveals untapped genetic potential in Africa’s indigenous cereal crop sorghum. Nat Commun 2013;4:2320.

[44] Ellegren H, Galtier N. Determinants of genetic diversity. Nat Rev Genet 2016;17:422–33.

[45] Kawahara Y, de la Bastide M, Hamilton JP, Kanamori H, McCombie WR, Ouyang S, et al. Improvement of the *Oryza sativa* Nipponbare reference genome using next generation sequence and optical map data. Rice 2013;6:4.

[46] Maccaferri M, Harris NS, Twardziok SO, Pasam RK, Gundlach H, Spannagl M, et al.

Durum wheat genome highlights past domestication signatures and future improvement targets. Nat Genet 2019;51:885–95.

[47] Chen J, Wang Z, Tan K, Huang W, Shi J, Li T, et al. A complete telomere-to-telomere assembly of the maize genome. Nat Genet 2023:1–11.

[48] Schnable PS, Ware D, Fulton RS, Stein JC, Wei F, Pasternak S, et al. The B73 maize genome: complexity, diversity, and dynamics. Science 2009;326:1112–5.

[49] THE INTERNATIONAL WHEAT GENOME SEQUENCING CONSORTIUM (IWGSC). A chromosome-based draft sequence of the hexaploid bread wheat (*Triticum aestivum*) genome. Science 2014;345:1251788.

[50] Murat F, Armero A, Pont C, Klopp C, Salse J. Reconstructing the genome of the most recent common ancestor of flowering plants. Nat Genet 2017;49:490–6.

[51] Shi T, Huneau C, Zhang Y, Li Y, Chen J, Salse J, et al. The slow-evolving *Acorus tatarinowii* genome sheds light on ancestral monocot evolution. Nat Plants 2022;8:764–77.

[52] Sun Y, Shang L, Zhu Q-H, Fan L, Guo L. Twenty years of plant genome sequencing: achievements and challenges. Trends Plant Sci 2022;27:391–401.

[53] Ffrench-Constant RH, Bass C. Does resistance really carry a fitness cost? Curr Opin Insect Sci 2017;21:39–46.

[54] Nelson R, Wiesner-Hanks T, Wisser R, Balint-Kurti P. Navigating complexity to breed disease-resistant crops. Nat Rev Genet 2018;19:21–33.

[55] Vila-Aiub MM, Yu Q, Powles SB. Do plants pay a fitness cost to be resistant to glyphosate? New Phytol 2019;223:532–47.

[56] He Z, Webster S, He SY. Growth–defense trade-offs in plants. Curr Biol

2022;32:R634–9.
[57] Mounger J, Ainouche ML, Bossdorf O, Cavé-Radet A, Li B, Parepa M, et al. Epigenetics and the success of invasive plants. Philos Trans R Soc B Biol Sci 2021;376:20200117.
[58] Belton J-M, McCord RP, Gibcus JH, Naumova N, Zhan Y, Dekker J. Hi–C: A comprehensive technique to capture the conformation of genomes. Methods 2012;58:268–76.
[59] Cheng H, Concepcion GT, Feng X, Zhang H, Li H. Haplotype-resolved *de novo* assembly using phased assembly graphs with hifiasm. Nat Methods 2021;18:170–5.
[60] Li H. Minimap2: pairwise alignment for nucleotide sequences. Bioinformatics 2018;34:3094–100.
[61] Vaser R, Sović I, Nagarajan N, Šikić M. Fast and accurate *de novo* genome assembly from long uncorrected reads. Genome Res 2017;27:737–46.
[62] Dudchenko O, Batra SS, Omer AD, Nyquist SK, Hoeger M, Durand NC, et al. *De novo* assembly of the *Aedes aegypti* genome using Hi-C yields chromosome-length scaffolds. Science 2017;356:92–5.
[63] Durand NC, Shamim MS, Machol I, Rao SSP, Huntley MH, Lander ES, et al. Juicer provides a one-click system for analyzing loop-resolution Hi-C experiments. Cell Syst 2016;3:95–8.
[64] Jia K-H, Wang Z-X, Wang L, Li G-Y, Zhang W, Wang X-L, et al. SubPhaser: a robust allopolyploid subgenome phasing method based on subgenome-specific k-mers. New Phytol 2022;235:801–9.

[65] Tarailo-Graovac M, Chen N. Using RepeatMasker to identify repetitive elements in genomic sequences. Curr Protoc Bioinforma 2009;25:4.10.1-4.10.14.

[66] Salamov AA, Solovyev VV. *Ab initio* gene finding in *Drosophila* genomic DNA. Genome Res 2000;10:516–22.

[67] Stanke M, Keller O, Gunduz I, Hayes A, Waack S, Morgenstern B. AUGUSTUS: *ab initio* prediction of alternative transcripts. Nucleic Acids Res 2006;34:W435–9.

[68] Haas BJ, Salzberg SL, Zhu W, Pertea M, Allen JE, Orvis J, et al. Automated eukaryotic gene structure annotation using EVidenceModeler and the Program to Assemble Spliced Alignments. Genome Biol 2008;9:R7.

[69] Zdobnov EM, Apweiler R. InterProScan – an integration platform for the signature-recognition methods in InterPro. Bioinformatics 2001;17:847–8.

[70] Zhang Y, Chu J, Cheng H, Li H. *De novo* reconstruction of satellite repeat units from sequence data 2023.

[71] Wang Z, Miao H, Liu J, Xu B, Yao X, Xu C, et al. *Musa balbisiana* genome reveals subgenome evolution and functional divergence. Nat Plants 2019;5:810–21.

[72] Zhao H, Wang S, Wang J, Chen C, Hao S, Chen L, et al. The chromosome-level genome assemblies of two rattans (*Calamus simplicifolius* and *Daemonorops jenkinsiana*). GigaScience 2018;7:giy097.

[73] Ma P-F, Liu Y-L, Jin G-H, Liu J-X, Wu H, He J, et al. The *Pharus latifolius* genome bridges the gap of early grass evolution. Plant Cell 2021;33:846–64.

[74] Sasaki T. The map-based sequence of the rice genome. Nature 2005;436:793–800.

[75] Price MN, Dehal PS, Arkin AP. FastTree 2 – Approximately Maximum-Likelihood

Trees for Large Alignments. PLOS ONE 2010;5:e9490.
[76] Letunic I, Bork P. Interactive tree of life (iTOL) v3: an online tool for the display and annotation of phylogenetic and other trees. Nucleic Acids Res 2016;44:W242–5.
[77] Minh BQ, Schmidt HA, Chernomor O, Schrempf D, Woodhams MD, von Haeseler A, et al. IQ-TREE 2: New models and efficient methods for phylogenetic inference in the genomic era. Mol Biol Evol 2020;37:1530–4.
[78] Yang Z. PAML 4: Phylogenetic analysis by maximum likelihood. Mol Biol Evol 2007;24:1586–91.
[79] Kumar S, Suleski M, Craig JM, Kasprowicz AE, Sanderford M, Li M, et al. TimeTree 5: An expanded resource for species divergence times. Mol Biol Evol 2022;39:msac174.
[80] Xu P, Xu J, Liu G, Chen L, Zhou Z, Peng W, et al. The allotetraploid origin and asymmetrical genome evolution of the common carp *Cyprinus carpio*. Nat Commun 2019;10:4625.
[81] Sun P, Jiao B, Yang Y, Shan L, Li T, Li X, et al. WGDI: A user-friendly toolkit for evolutionary analyses of whole-genome duplications and ancestral karyotypes. Mol Plant 2022;15:1841–51.
[82] Zhang Z, Li J, Zhao X-Q, Wang J, Wong GK-S, Yu J. KaKs_Calculator: Calculating Ka and Ks through model selection and model averaging. Genomics Proteomics Bioinformatics 2006;4:259–63.
[83] Bennetzen JL, Schmutz J, Wang H, Percifield R, Hawkins J, Pontaroli AC, et al. Reference genome sequence of the model plant *Setaria*. Nat Biotechnol 2012;30:555–61.
[84] Wu D, Shen E, Jiang B, Feng Y, Tang W, Lao S, et al. Genomic insights into the

evolution of *Echinochloa* species as weed and orphan crop. Nat Commun 2022;13:689.

[85] Hazzouri KM, Gros-Balthazard M, Flowers JM, Copetti D, Lemansour A, Lebrun M, et al. Genome-wide association mapping of date palm fruit traits. Nat Commun 2019;10:4680.

[86] Patel RK, Jain M. NGS QC Toolkit: A Toolkit for Quality Control of Next Generation Sequencing Data. PLOS ONE 2012;7:e30619.

[87] Ye C-Y, Tang W, Wu D, Jia L, Qiu J, Chen M, et al. Genomic evidence of human selection on Vavilovian mimicry. Nat Ecol Evol 2019;3:1474–82.

[88] Zheng X, Levine D, Shen J, Gogarten SM, Laurie C, Weir BS. A high-performance computing toolset for relatedness and principal component analysis of SNP data. Bioinformatics 2012;28:3326–8.

[89] Jin J-J, Yu W-B, Yang J-B, Song Y, dePamphilis CW, Yi T-S, et al. GetOrganelle: a fast and versatile toolkit for accurate de novo assembly of organelle genomes. Genome Biol 2020;21:241.

[90] Tillich M, Lehwark P, Pellizzer T, Ulbricht-Jones ES, Fischer A, Bock R, et al. GeSeq – versatile and accurate annotation of organelle genomes. Nucleic Acids Res 2017;45:W6–11.

[91] Katoh K, Standley DM. MAFFT Multiple Sequence Alignment Software Version 7: Improvements in Performance and Usability. Mol Biol Evol 2013;30:772–80.

[92] Huang Y, Guo l, Xie l, Shang N, Wu D, Ye C, et al. Supporting data for "A reference genome of Commelinales provides insights into the commelinids evolution and global spread of water hyacinth (Pontederia crassipes)" GigaScience Database. 2024. http://doi.org/10.5524/102495.

Table 1 Summary of *P. crassipes* plant materials collection, genome sequencing and annotation by this study.

Items	Data	
**Reference genome**		
Plant material	Zijingang#1 from Hangzhou, China	
Estimated genome size, Mb	1,278	
Sequencing platform (genome coverage)	Pacbio HiFi (52×) + Illumina (61×) + HiC (100×)	
Assembly size, Mb	1,220	
Scaffold N50, Mb	77.2	
Number of genes annotated	65,299	
BUSCO assessment, %	95.2%	
Subgenome	Sub-A	Sub-B
Assembly size, Mb	640.2	577.6
Number of genes annotated	33,608	31,691
BUSCO assessment, %	88.3%	86.5%
Percentage of repeat elements, %	58.61 %	52.92%
**Population investigation**		
Sequencing platform (genome coverage)	Illumina (36×)	
Number of collection locations	9	
Country sampled	Brazil (5), China (1), Malaysia (1), Germany (2)	

Legends of figures

Figure 1. Phylogeny and evolution of *P. crassipes* genome. **A** Single-copy gene based ultrametric phylogenetic tree and divergence times of *P. crassipes* and other representative species of the commelinids with *A. tatarinowii* as an outgroup. **B**, Distribution of synonymous substitution per site (K_S) of paralogue genes in collinear regions of *P. crassipes* and orthologous genes between *P. crassipes* and other members of the commelinids (*C. nucifera*, *C. simplicifolius*, *Z. officinale*, *M. balbisiana*, *P. latifolius* and *A. tatarinowii*.) **C**, Dot plots showing the conserved genomic synteny between *P. crassipes* and *C. nucifera*. An example of conserved synteny region originated from the τ WGD event is marked in rectangular.

Figure 2 Changes of gene family size during genome polyploidization of *P. crassipes*.

A, Dot matrix plot and distribution of fold changes of gene family sizes in *P. crassipes* compared with diploid *O. sativa* and tetraploid *E. oryzicola*. Regarding the distribution of gene family sizes (subfigures at lower right corner), the highest percentage was observed in the gene families of *P. crassipes* that were two times bigger in size than those of *O. sativa* (left) and the same size as those of *E. oryzicola* (right). **B,** Comparison of disease-resistance -related gene family sizes between *P. crassipes* and other commelinids species. + and − indicate increase and decrease in size, respectively, relative to *P. crassipe*s. $*P < 0.01$, $**P < 0.001$, $***P < 0.0001$, Fisher's exact test. **C-E**, Synteny retention ratio of different paralogous pairs in ten gene families after polyploidization of *P. crassipes*. The graphs show the percentage of retained gene pairs experienced the two polyploidization events (C, A1:A2:B1:B2 = 1:1:1:1), one of the two events (D, A1:A2 or B1:B2 = 1:1), or two subgenomes (E, subA:subB = 1:1). The dashed lines represent the average retention ratio of genes across the genome. LLD: legume lectin domain.

Figure 3 Inference of proto-chromosomes and ancestral karyotypes of the commelinids. **A**, Identification of proto-chromosomes based on synteny regions among extant chromosomes. Alignments between proto- and extant chromosomes shown in different colors indicate the different origination from the proto-chromosomes. Cn: *C. nucifera*; Ac: *A. comosus*; Pc: *P. crassipes*. **B**, Reconstruction of ancestral karyotypes and their phylogeny of the commelinids. Ancestral chromosomes at specific evolutionary nodes were inferred and denoted with different colors. Whole genome duplication and triplication events are shown in red and blue circles, respectively.

Figure 4 Genetic diversity and phylogeny of global *P. crassipes*. **A**, The collection locations of the ten water hyacinth lines used in this study are indicated by circles and the chloroplast genomes (A and B) are labeled with two different colors. **B**, A phylogenetic tree of the nine lines built based on their nuclear genomic SNPs relative to the reference Zijingang genome.

769

Click here to access/download;Figure;01_Phylogany_WGDsamll.pdf

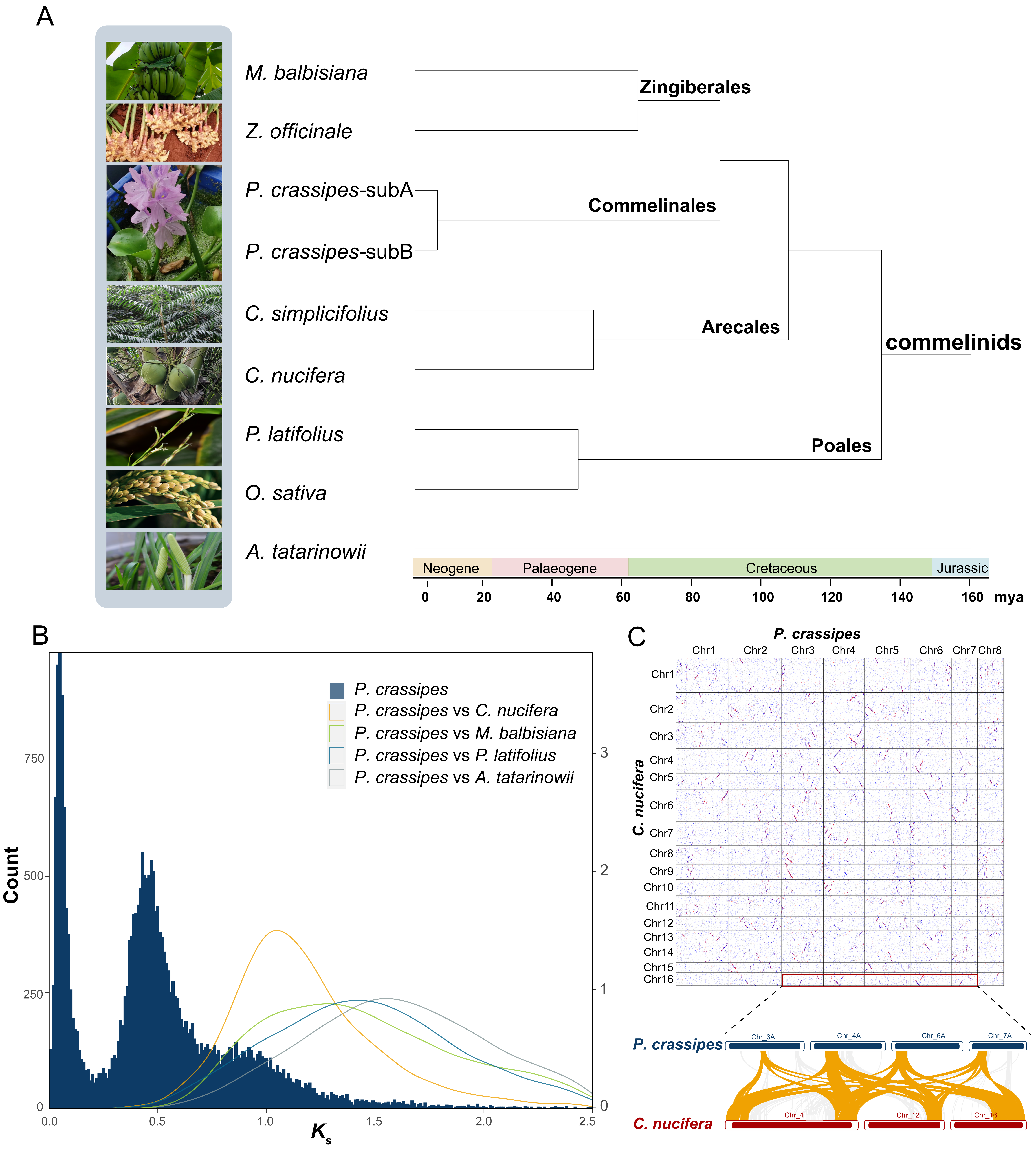

Figure2

Click here to access/download;Figure;02_family_size_ABC.pdf

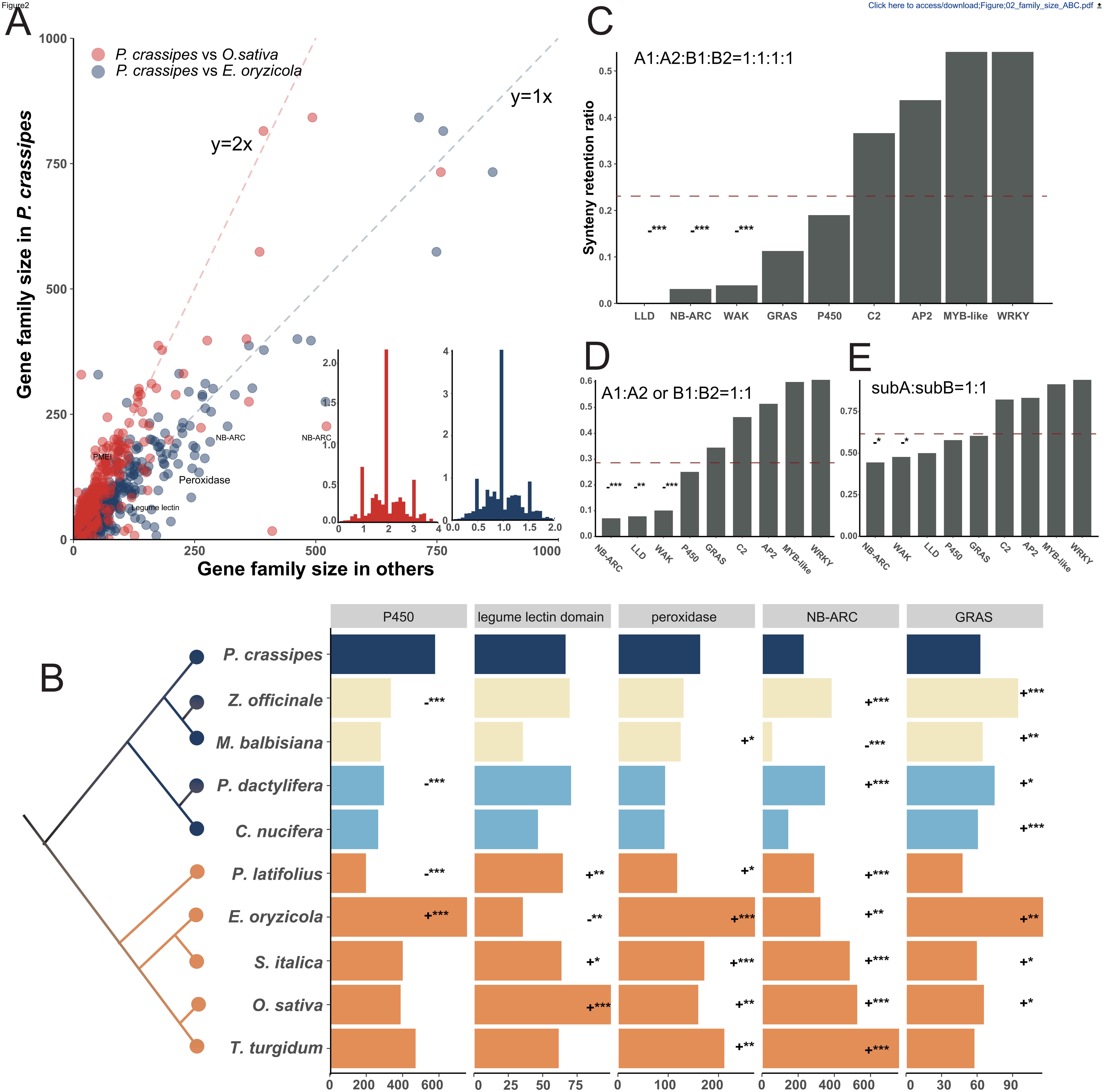

Click here to access/download;Figure;03_ancestral_final.pdf

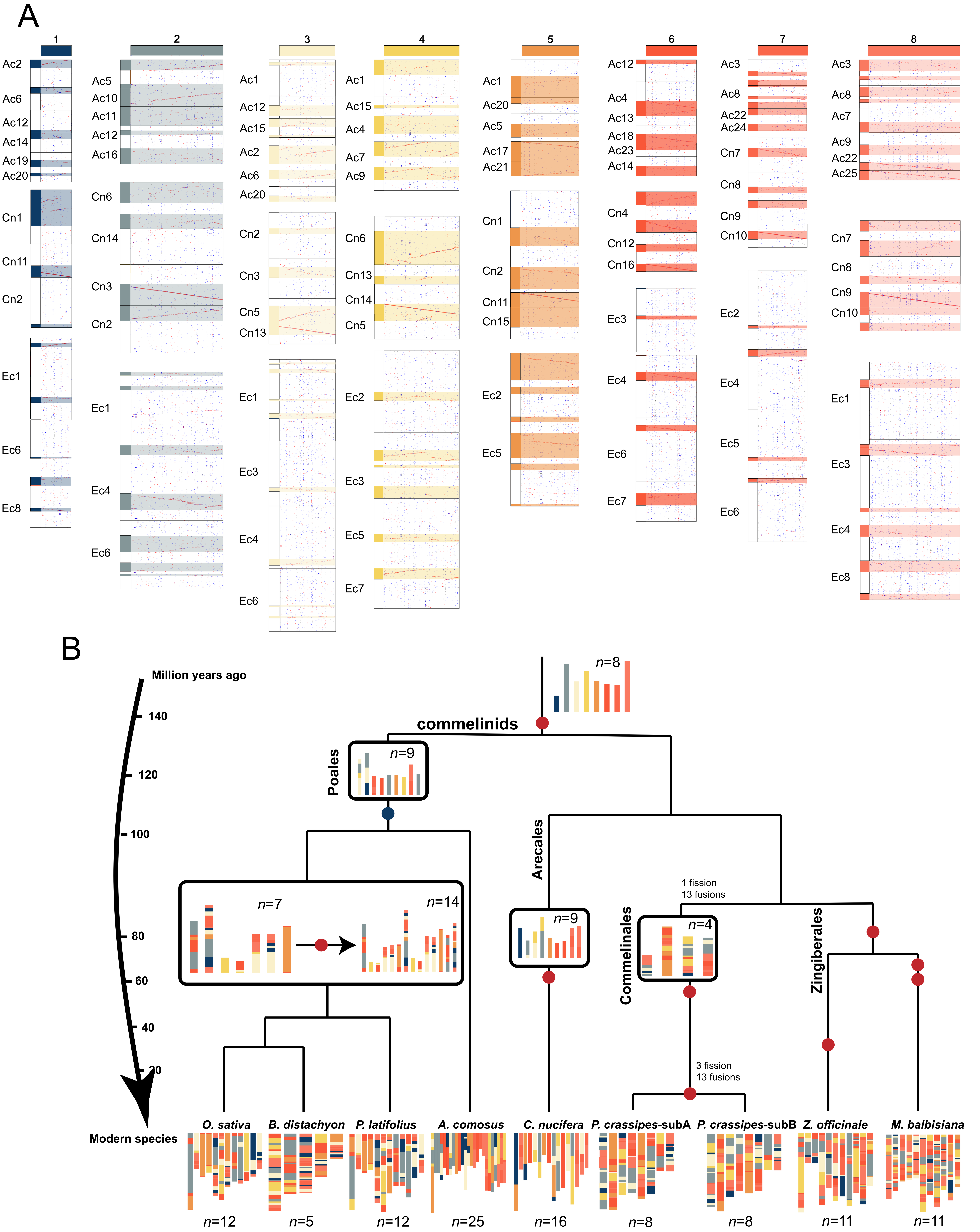

Figure4
Click here to access/download;Figure;04_chlomaptree.pdf

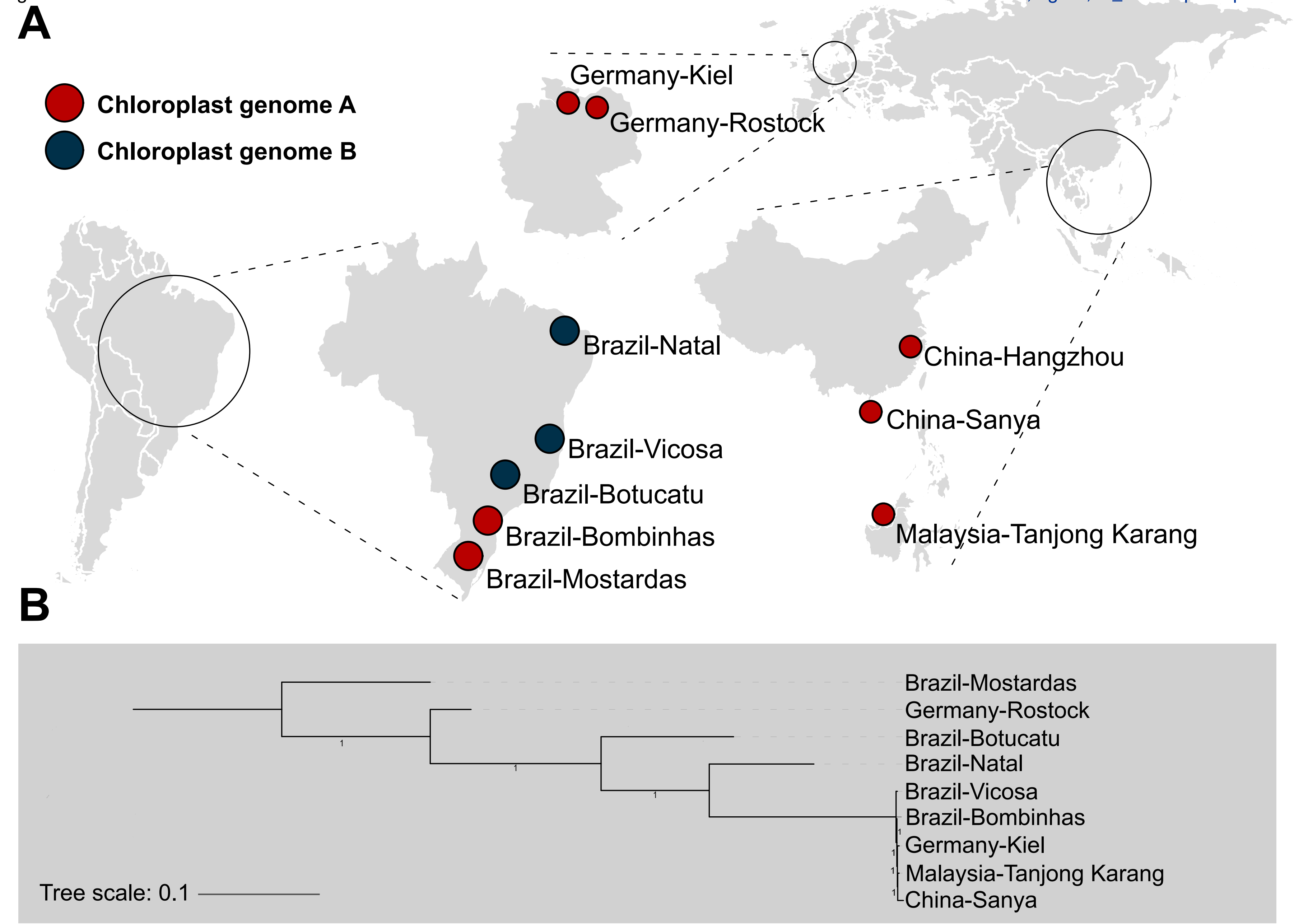

Click here to access/download
Supplementary Material
Sup_Fig1.pdf

Click here to access/download
Supplementary Material
Sup_Fig2.pdf

Click here to access/download

Supplementary Material

Sup_Fig3.pdf

Click here to access/download
Supplementary Material
Sup_Fig4.pdf

Click here to access/download
Supplementary Material
Sup_Fig5.pdf

Click here to access/download
Supplementary Material
Sup_Fig6 .pdf

Click here to access/download
Supplementary Material
Sup_Fig7.pdf

Click here to access/download
Supplementary Material
Sup_Fig8.pdf

Cover letter

Click here to access/download;Personal Cover;cover_letter-Ecra_genome_final.docx

2023-08-30

Dear Editor,

Please find our manuscript titled "**A reference genome of Commelinales provides insights into the commelinids evolution and global spread of water hyacinth (*Eichhornia crassipes*)**" for publication in **GigaScience.**

Commelinales belongs to the commelinids clade which also comprises Poales that includes the most important monocot species, such as rice, wheat, and maize. No reference genome within the Commelinales order has been generated up to now, which has hindered the elucidation of the phylogenetic puzzle of commelinids. Water hyacinth (*Eichhornia crassipes*), a member of Commelinales, is one of the devastating aquatic weeds. Here, we present a chromosome-scale reference genome of the tetraploid water hyacinth with a total length of 1.22 Gb (over 95% of the estimated size) across eight pseudochromosome pairs. With the representative genomes, we reconstructed phylogeny of the commelinids, which supported Zingiberales and Commelinales being sister lineages of Arecales. We also reconstructed ancestral karyotypes of the commelinids clade and confirmed the ancient commelinids genome having eight chromosomes but not five as previously reported. Contraction of disease-resistance genes during polyploidization of water hyacinth was revealed, likely a result of fitness requirement for its role as a weed. Genetic diversity analysis using nine water hyacinth lines from three continents (South America, Asia and Europe) revealed very closely related nuclear genomes and almost identical chloroplast genomes of the materials. It demonstrated that the global water hyacinths have a common origin in Brazil. The genomic resources of *E. crassipes* reported here contribute a crucial missing link of the commilinids species and offer novel insights into their phylogeny.

In short, this work reported a high-quality reference genome of *Eichhornia crassipes* (water hyacinth), the first for the order of Commelinales, confirmed the ancestral karyotypes of the commelinids with eight chromosomes and demonstrated a global spread event of *E. crassipes* from Brazil.

This manuscript has not been published or presented elsewhere in part or in entirety and is not under consideration by another journal. We have read and understood your journal's policies, and we believe that neither the manuscript nor the study violates any of these. There are no conflicts of interest to declare.

Please consider, as potential referees:

Yuannian Jiao
Expert of plant genomics and evolution
Institute of Botany, Chinese Academy of Sciences
jiaoyn@ibcas.ac.cn

Sanwen Huang
Expert of plant genomics
Chinese Academy of Tropical Agriculture Science
huangsanwen@caas.cn

Xuehui Huang
Expert in plant genomics
Shanghai Normal University, China
xhhuang@shnu.edu.cn

Toshiyuki Imaizumi
Expert in weed science and genome
National Agriculture and Food Research Organization (NARO), Japan
toima@affrc.go.jp

Excluded referees for potential research competition:

Jin-Ming Chen and Qingfeng Wang
Wuhan Botanical Garden, Chinese Academy of Sciences
Todd Gaines
Colorado State University, USA

We believe that this paper will be of interest to the readership of your journal and hope you can consider it. I look forward to hearing from you soon.

Sincerely,

Longjiang Fan
Institute of Crop Science & Institute of Bioinformatics, Zhejiang University, China
Tel: 0086-0571-88982730
E-mail: fanlj@zju.edu.cn
