# Supplementary figures and images for: A reference genome of Commelinales provides insights into the commelinids evolution and global spread of water hyacinth (Pontederia crassipes)
Source: Gigascience. 2024 Mar 14;13:giae006. doi: 10.1093/gigascience/giae006 (PMC10938897; doi:10.1093/gigascience/giae006)

# A

## GenomeScope Profile

len:1,058,520,254bp uniq:31.2% het:0.757% kcov:27 err:0.173% dup:1.89% k:17

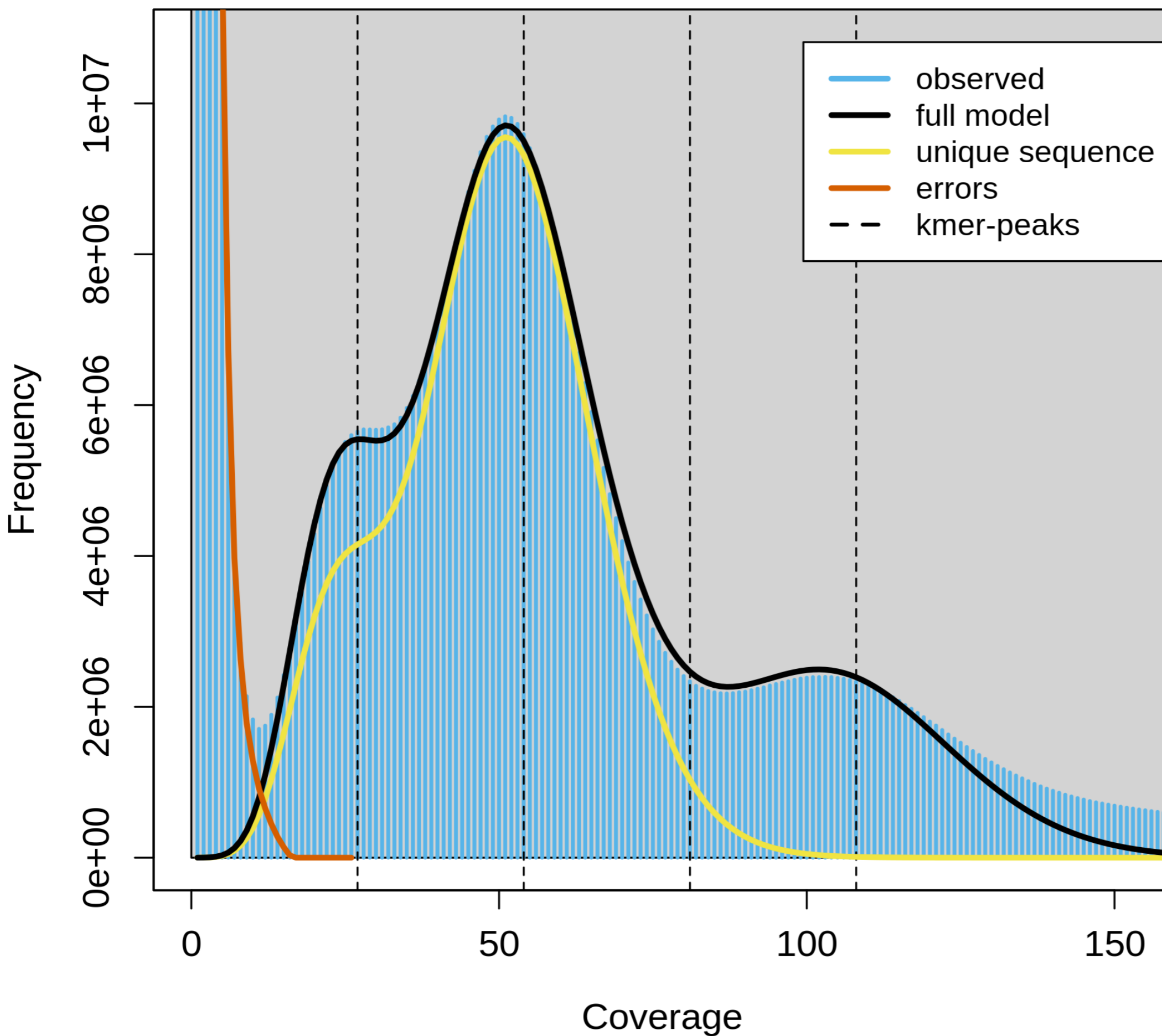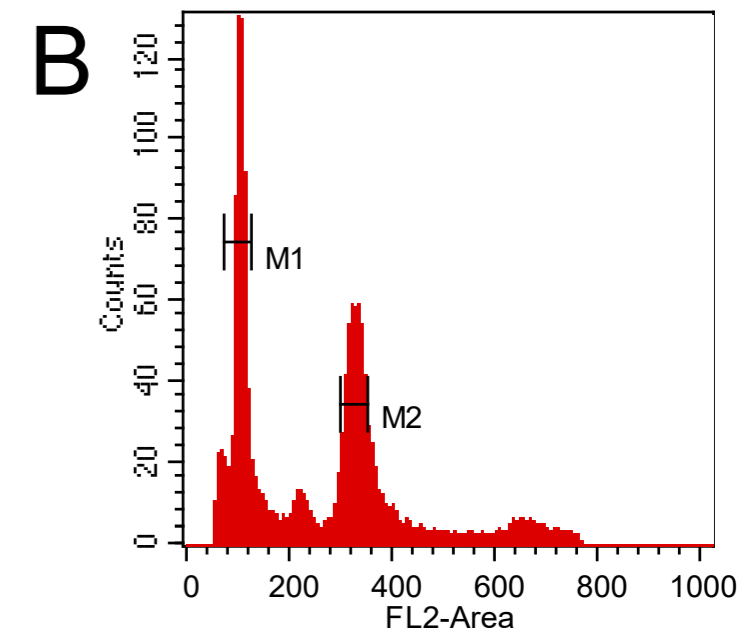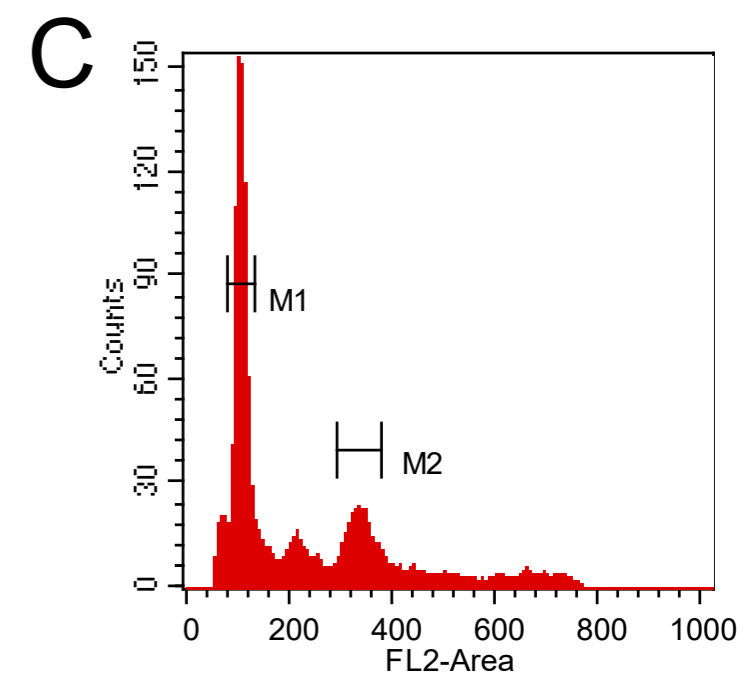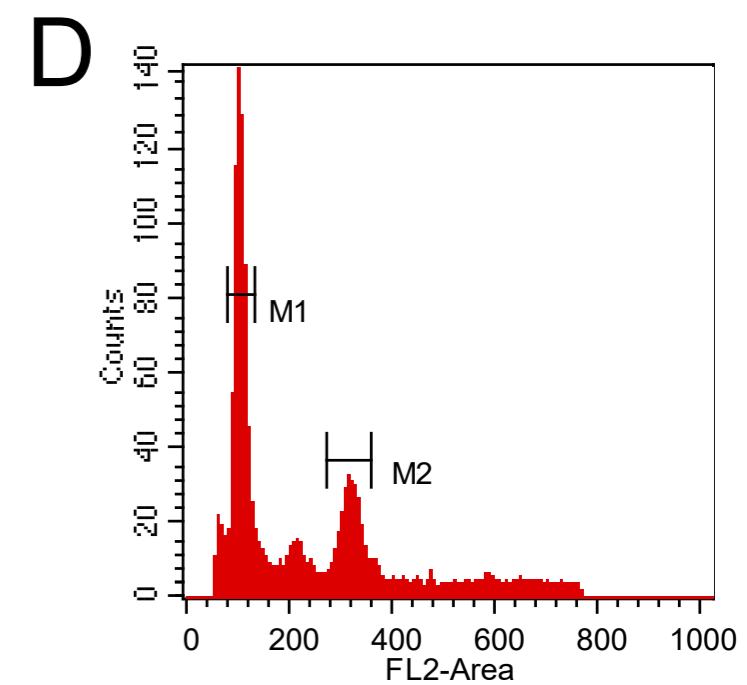

Supplement: giae006_Supplemental_Files [file giae006_supplemental_files.zip › Sup_Fig1.pdf]

A

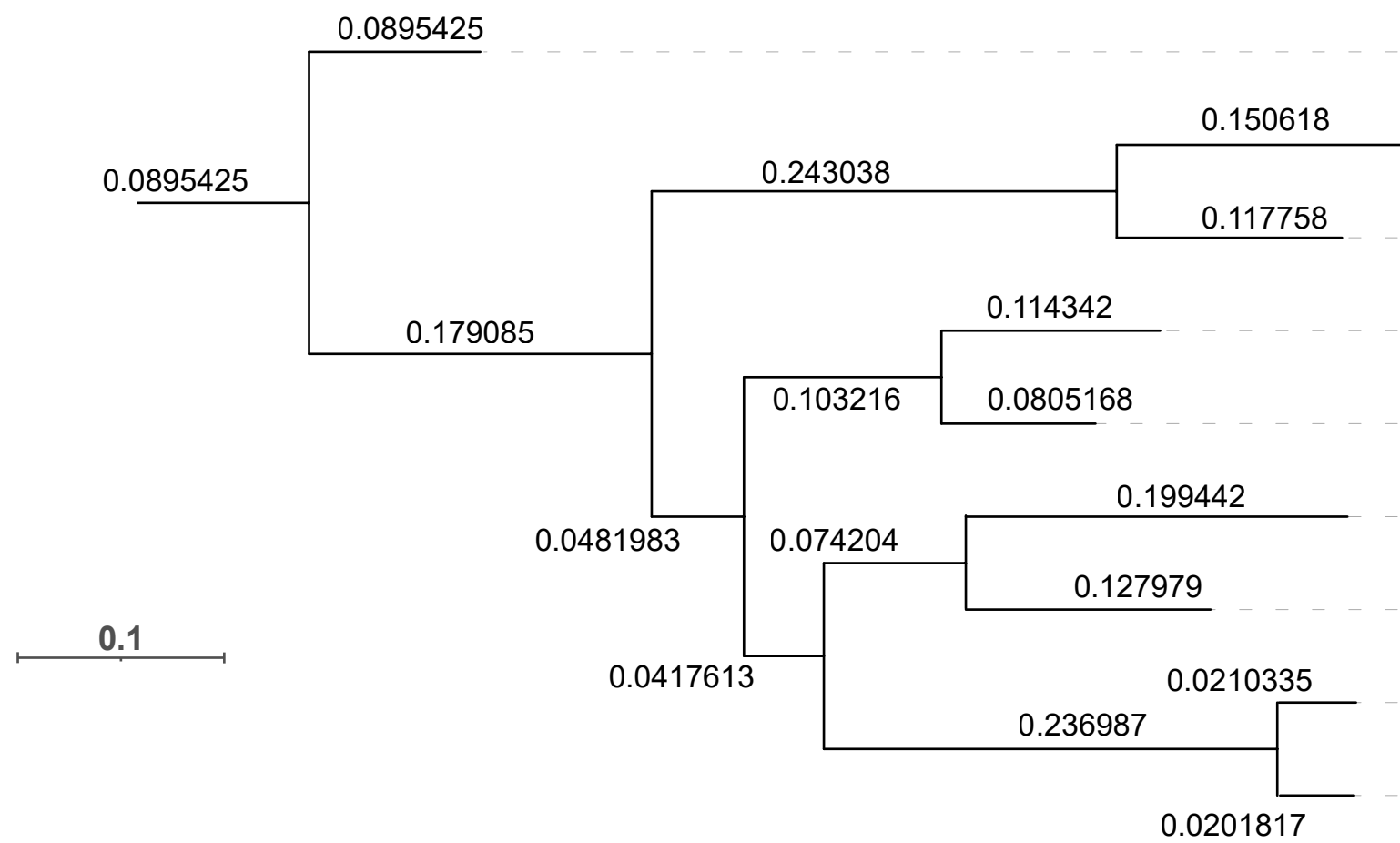

B

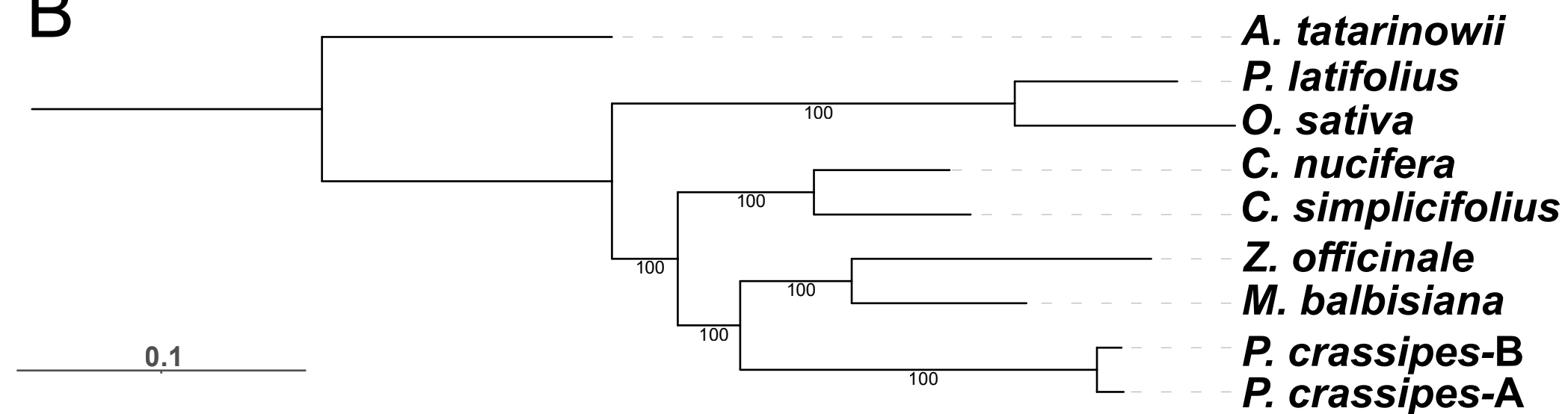

C

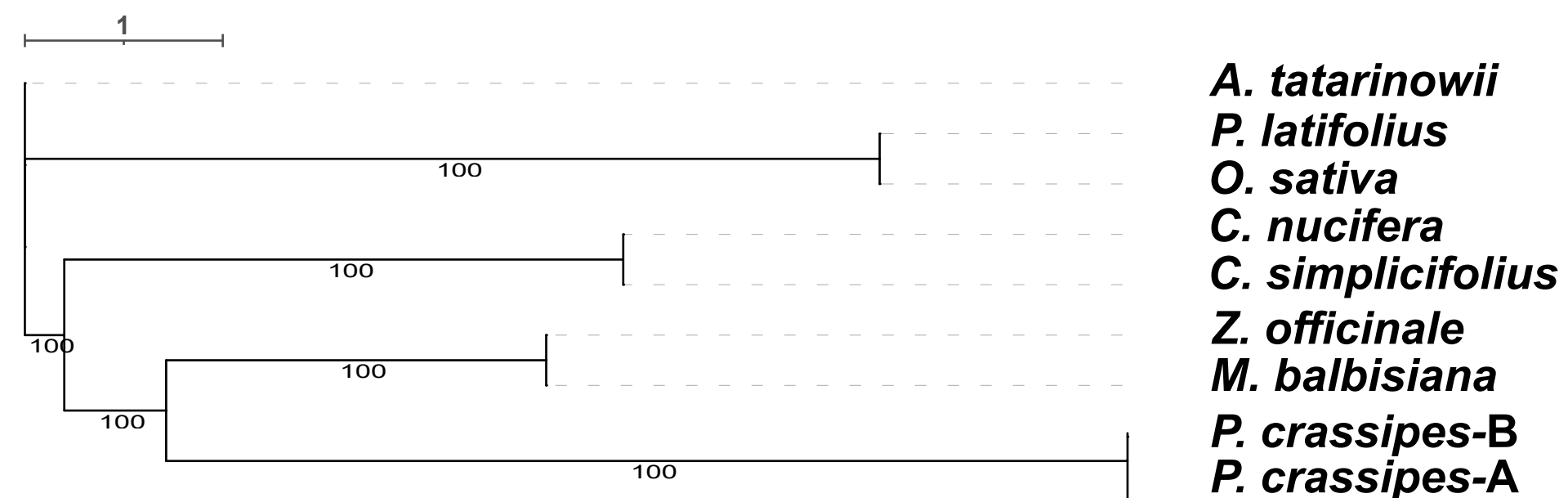

D

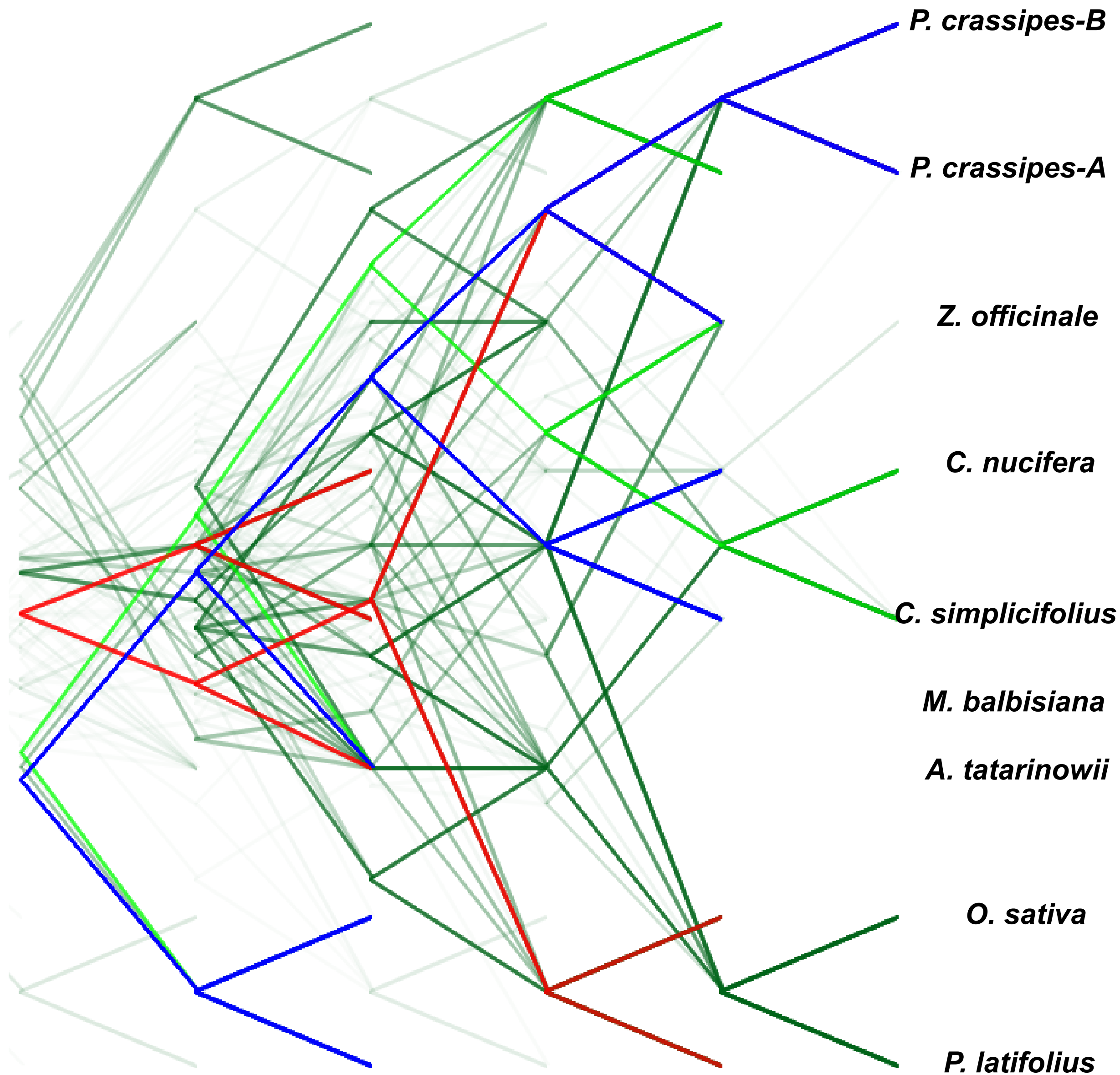

Supplement: giae006_Supplemental_Files [file giae006_supplemental_files.zip › Sup_Fig3.pdf]

# HLW

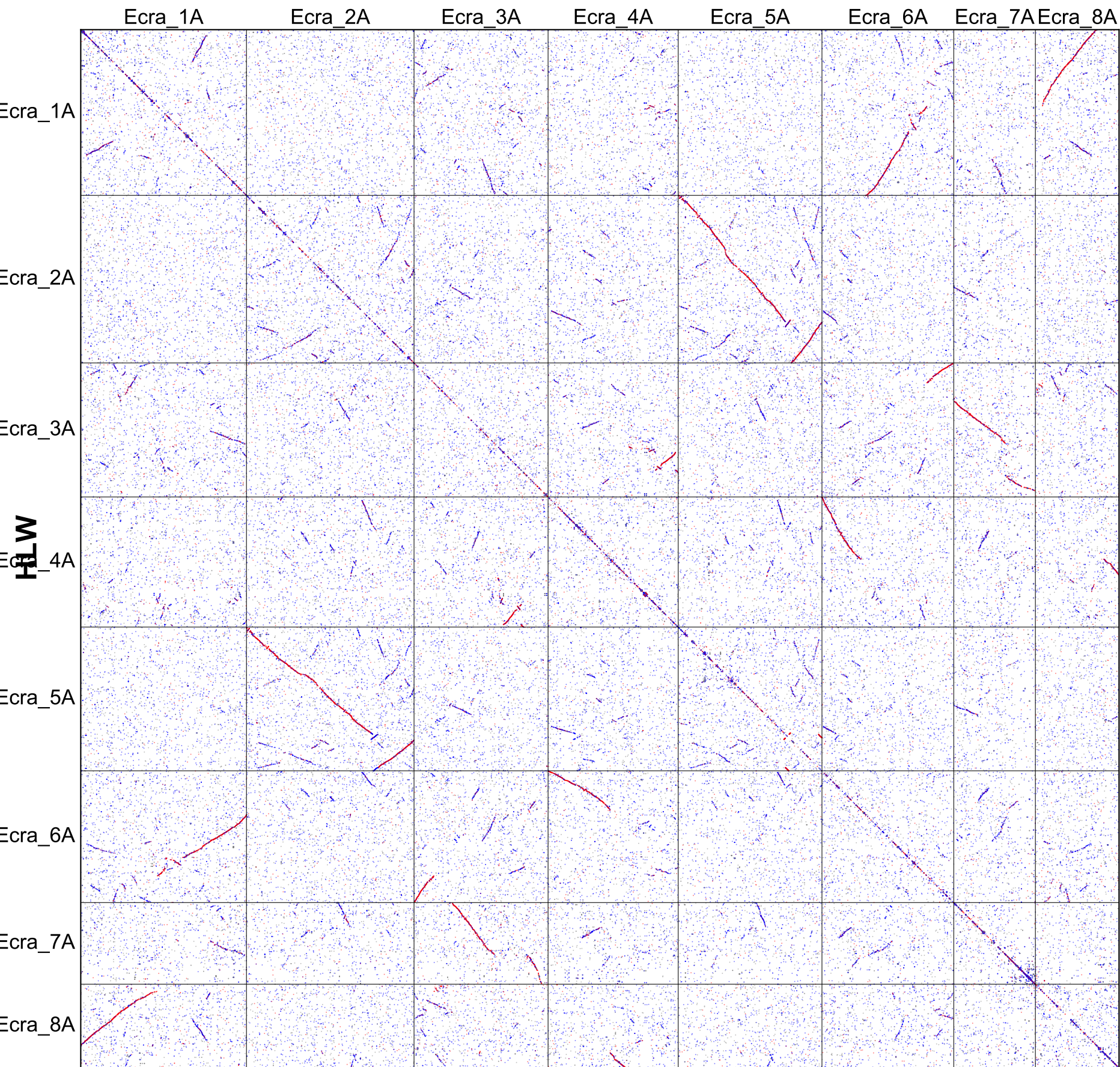

Supplement: giae006_Supplemental_Files [file giae006_supplemental_files.zip › Sup_Fig4.pdf]

# Compare between *C. nucifera* and *P. crassipes*

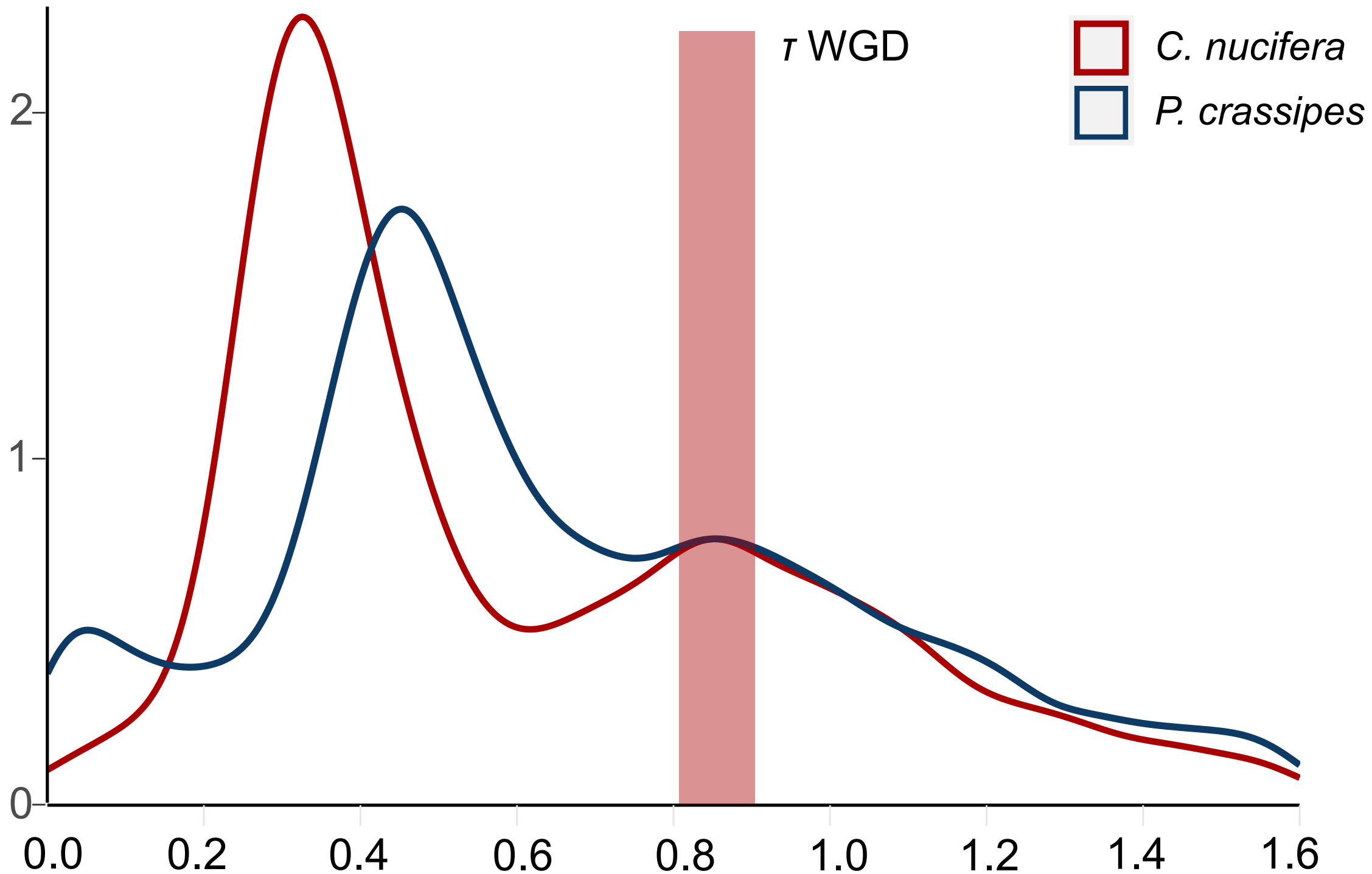

Supplement: giae006_Supplemental_Files [file giae006_supplemental_files.zip › Sup_Fig5.pdf]

Distribution of TE divergence

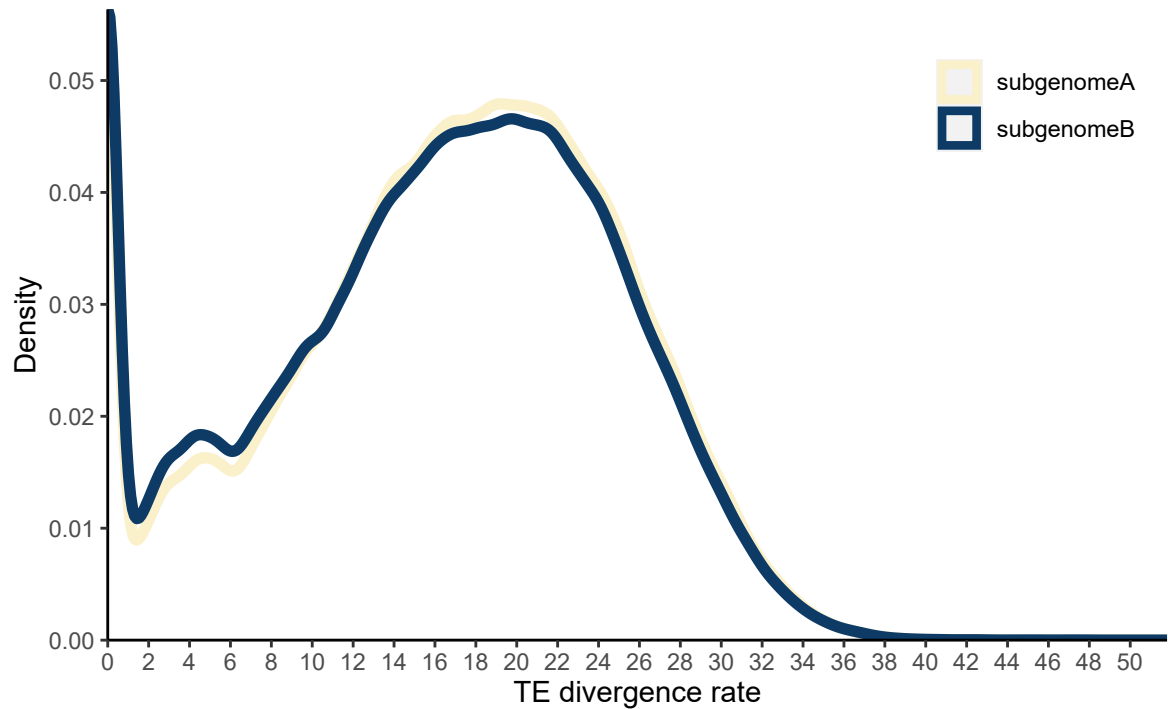

Supplement: giae006_Supplemental_Files [file giae006_supplemental_files.zip › Sup_Fig6 .pdf]

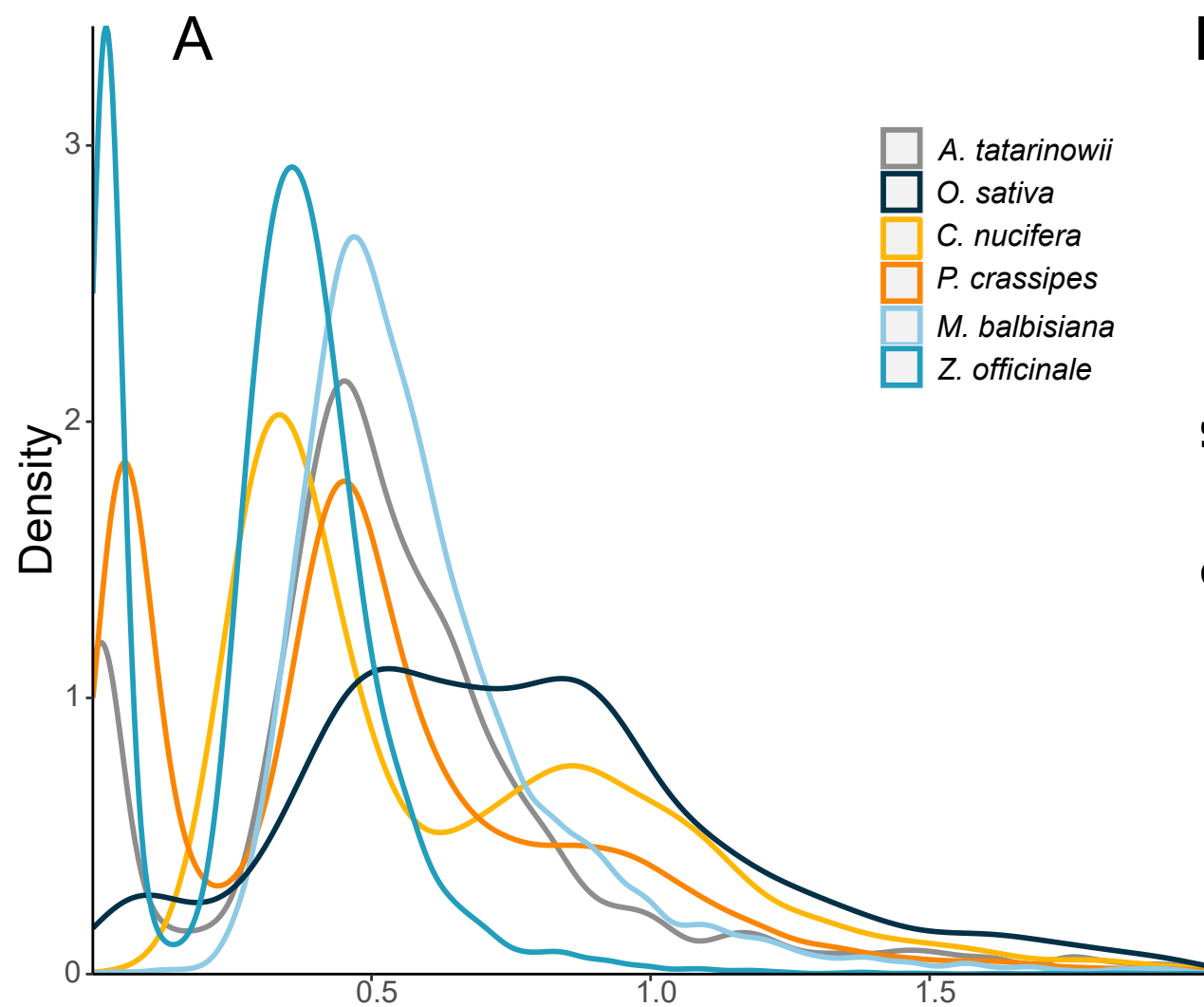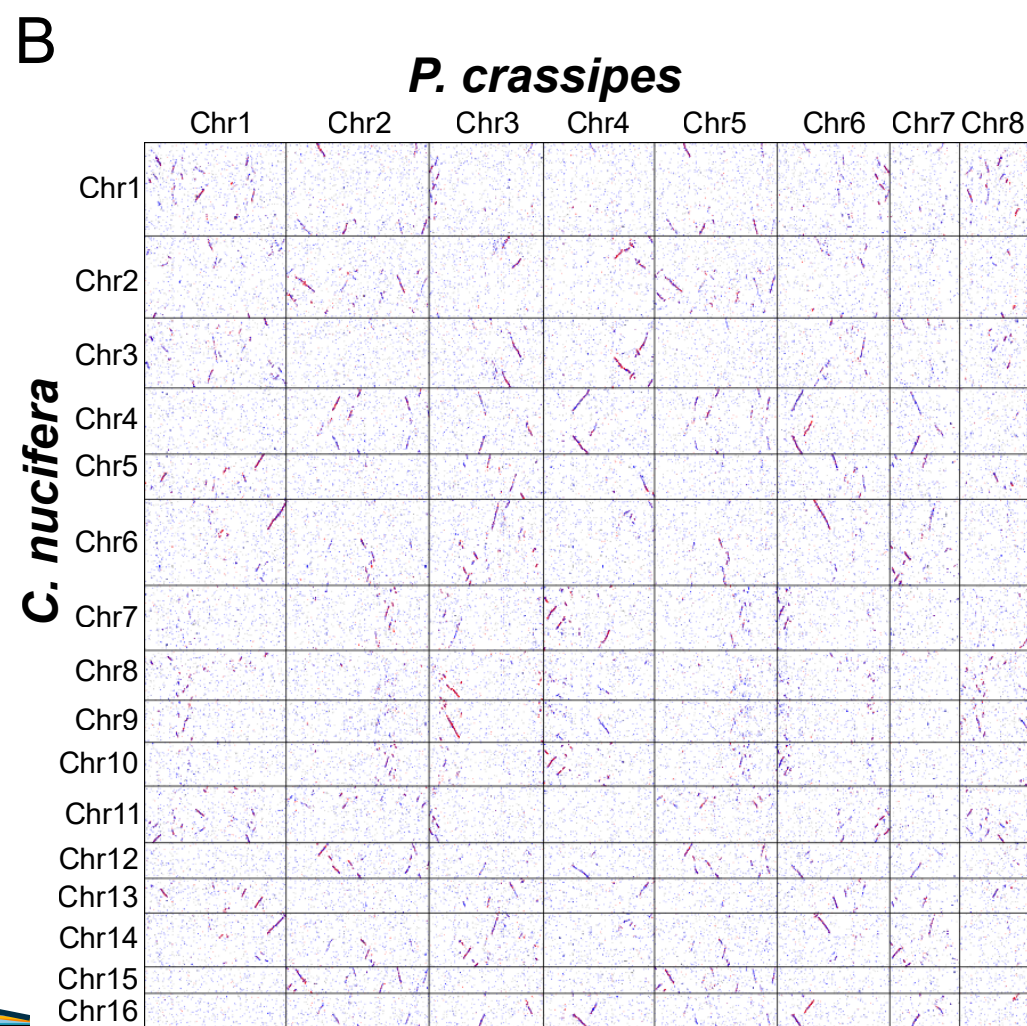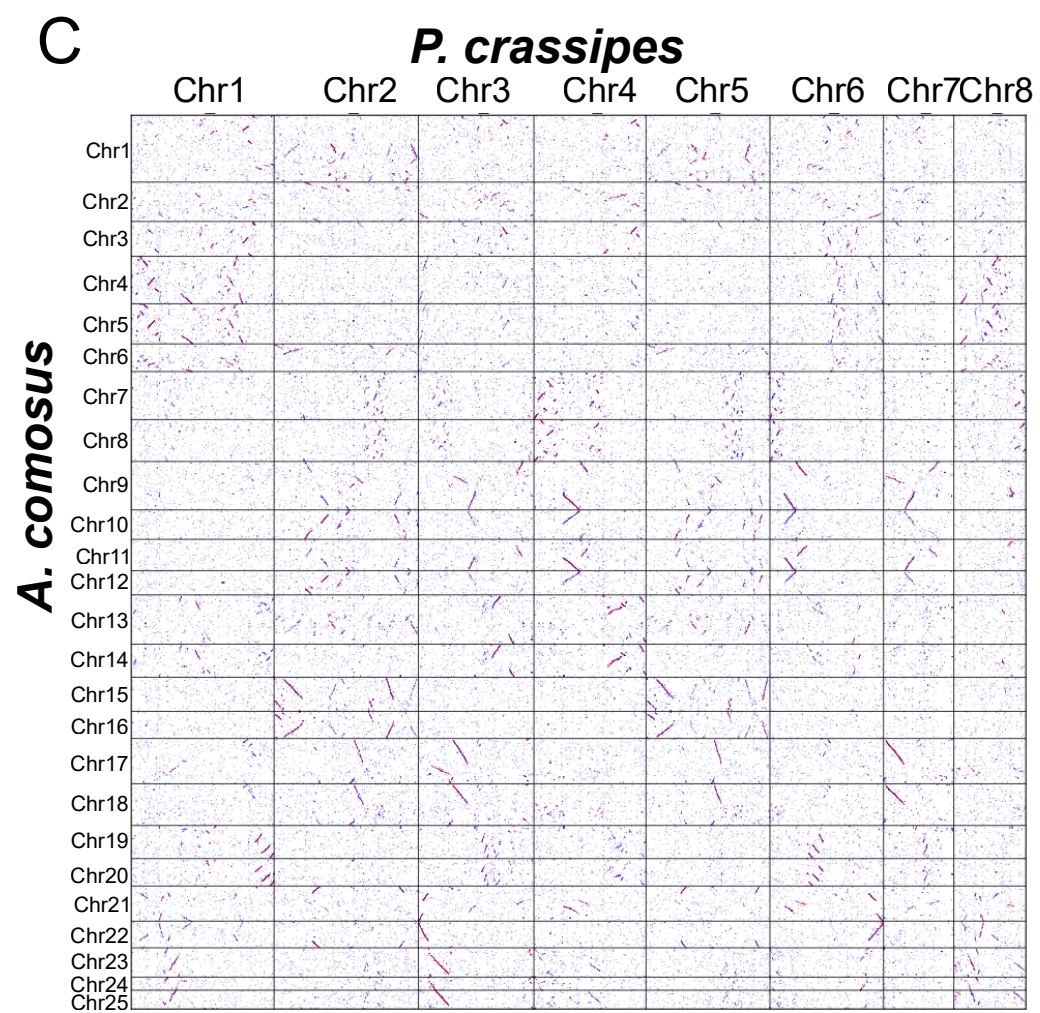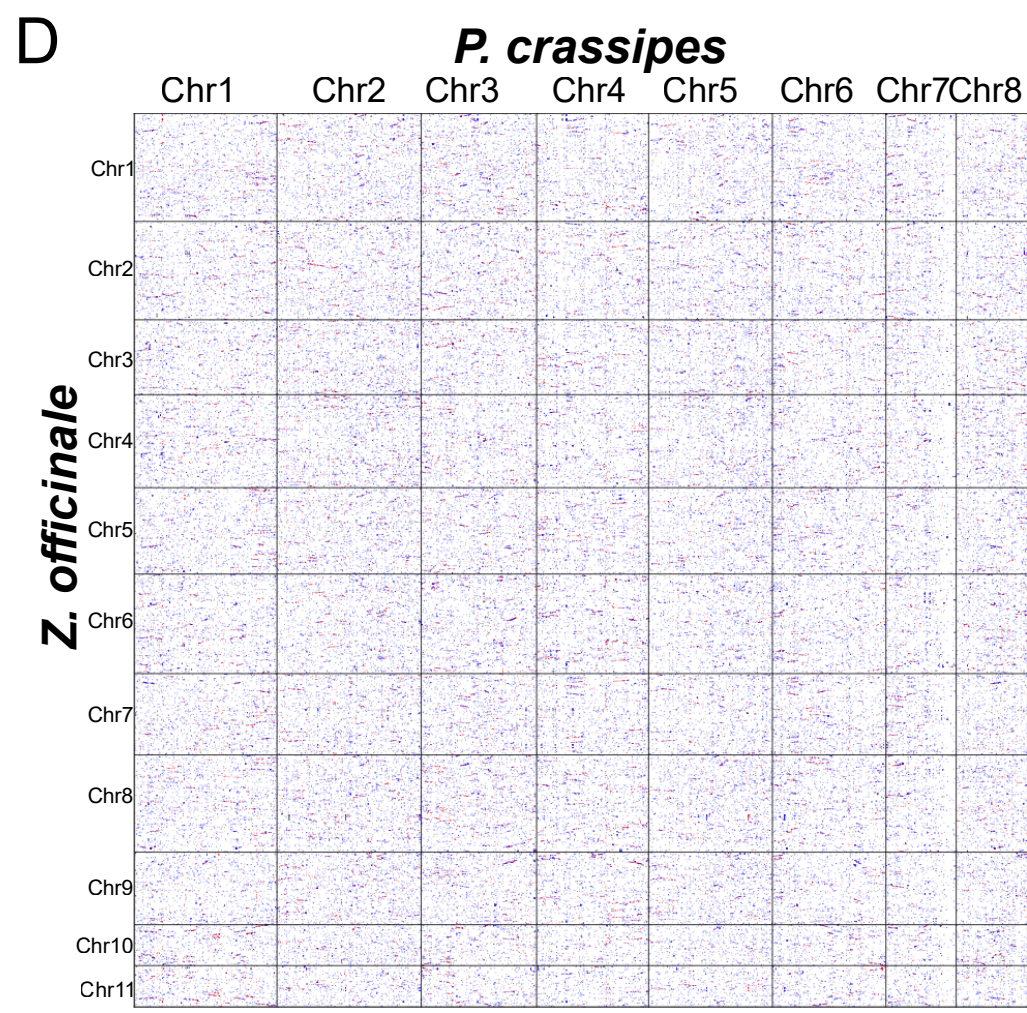

Supplement: giae006_Supplemental_Files [file giae006_supplemental_files.zip › Sup_Fig7.pdf]

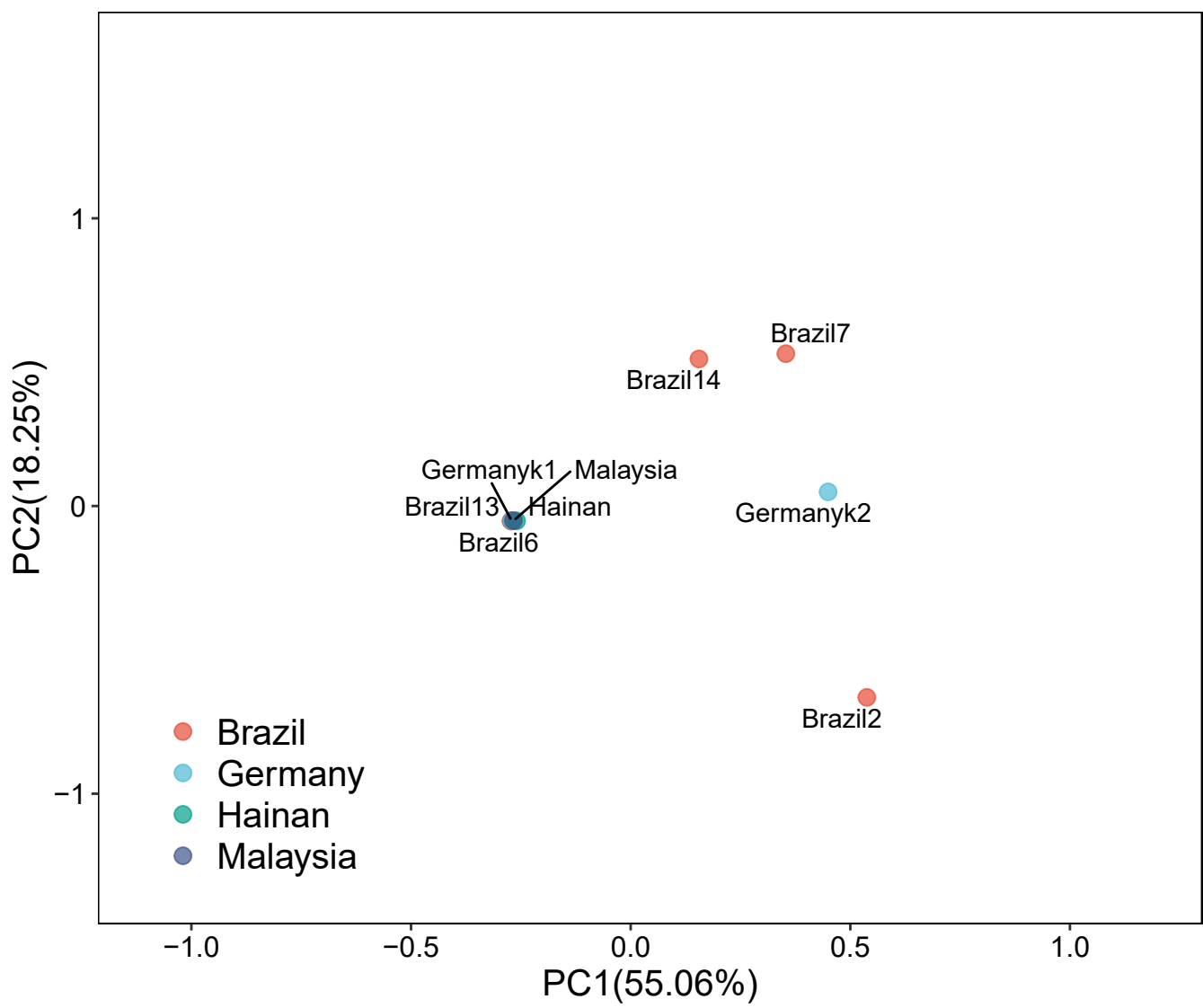

Supplement: giae006_Supplemental_Files [file giae006_supplemental_files.zip › Sup_Fig8.pdf]
