# Supplementary material for: A reference genome of Commelinales provides insights into the commelinids evolution and global spread of water hyacinth (Pontederia crassipes)
Source: Gigascience. 2024 Mar 14;13:giae006. doi: 10.1093/gigascience/giae006 (PMC10938897; doi:10.1093/gigascience/giae006)
Supplement: giae006_Supplemental_Files [file Sup_Fig2.pdf › Sup_Fig2.pdf]

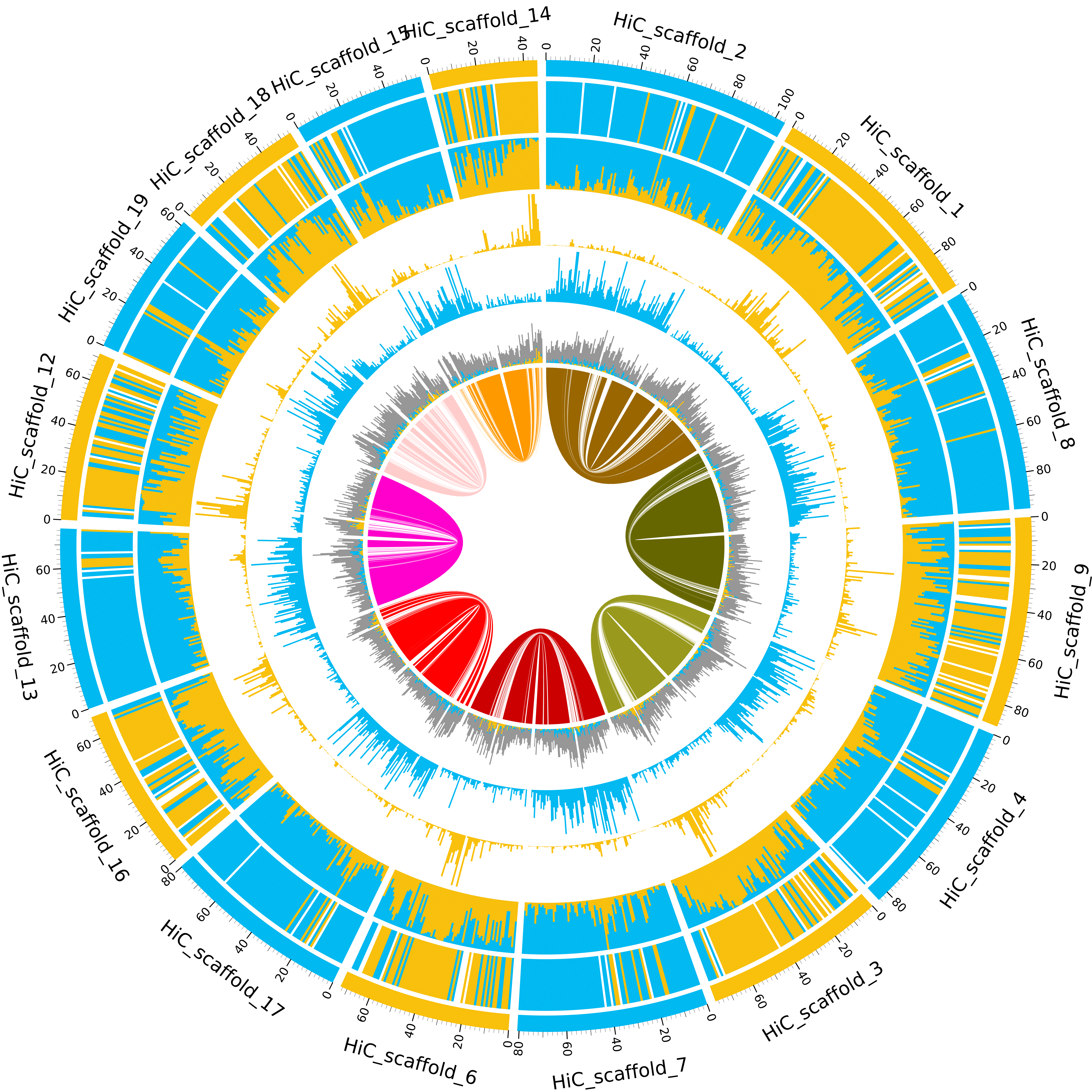
HiC_scaffold_14
HiC_scaffold_2
HiC_scaffold_1
HiC_scaffold_8
HiC_scaffold_9
HiC_scaffold_4
HiC_scaffold_3
HiC_scaffold_7
HiC_scaffold_6
HiC_scaffold_17
HiC_scaffold_16
HiC_scaffold_13
HiC_scaffold_12
HiC_scaffold_19
HiC_scaffold_18
HiC_scaffold_15
