# Supplementary material for: A reference genome of Commelinales provides insights into the commelinids evolution and global spread of water hyacinth (Pontederia crassipes)
Source: Gigascience. 2024 Mar 14;13:giae006. doi: 10.1093/gigascience/giae006 (PMC10938897; doi:10.1093/gigascience/giae006)
Supplement: giae006_Response_to_Reviewer_Comments_Original_Submission [file giae006_response_to_reviewer_comments_original_submission.pdf]

# Author's Response To Reviewer Comments

MS# GIGA-D-23-00274

TITLE: A reference genome of Commelinales provides insights into the commelinids evolution and global spread of water hyacinth (Pontederia crassipes)

Dear Editor in Chief:\\

Thank you very much for giving us an opportunity to revise our manuscript. We have carefully considered all of the comments and concerns raised by the two reviewers. Below you will find a point-by-point responses to the Reviewers' comments. We hope that we have fully addressed your and the Reviewers' concerns and that the substantially revised version of the manuscript is now suitable for publication in GigaScience.
Thank you for your time and effort on handling our manuscript!

Sincerely,

Longjiang Fan

........................................................................................................
Reviewer #1:
[The authors used 180 genes to infer the phylogeny of eight species, but the information about alignment, model test, bootstrap etc. are missing. It's not clear how did the authors generate the "orthologs-based tree". Did the author take it from OrthoFinder? Then the author built coalescent and concatenate trees and compared them with "orthologs-based tree". In general, both coalescent and concatenate trees are also orthologs-based tree. It seems that the authors compared the trees generated by different ML-based software (IQtree and RaxML) using the same dataset (180 orthologs). This doesn't say much about the robustness of the phylogeny but only to test the consistence between different software.]

>>Response: Sorry for the missing information and we have added the your requested information in the revised manuscript. In brief, the initial orthologs-based tree was generated directly by fasttree built-in OrthoFinder. To illustrate the robustness of this topology based on orthologs genes, we reconstructed the coalescent and concatenate trees using IQtree and RaxML, respectively, with all bootstrap values 100. Also, we generated a large number of trees representing all possible topologies using densitree to exclude interference from gene flow. These results are all consistent, suggesting a reliable result based on the 180 nuclear genes.

[The authors included 10 lines to infer the global origin and dispersal of water hyacinth and concluded that "water hyacinth originates mainly from a single dispersal event from Brazil". Firstly, the sampling size is too small to derive such conclusion. Secondly, the chloroplast genomes are almost identical, with only a 1bp indel, which doesn't provide much information about phylo-geographic history. Thirdly, in the phylogeny (figure 4b), a Brazil line was put at the root. What's the justification? At least an outgroup is needed to root the tree. In addition, information about the chloroplast genome assembly, SNP calling, phylogeny inference etc. are missing.]

>>Response: We agree your point about sampling size and our conclusion about water hyacinth's dispersal. We are collecting more water hyacinth globally and plan to do a global population investigation by genome re-sequencing. The water hyacinth is an famous invasive plant, which happened about one hundred years ago from south America and it will be interesting to observe genomic changes during the 100 years global dispersal. The 10 lines used in this study, we mainly used to estimate genetic diversity of water hyacinth. We have changed our statement about the conclusion of water hyacinth dispersal in the revised version.

We agree your point about the outgroup of phylogeny. As previously stated in our manuscript, the absence of genomic data on closely related Commelinales restricted us to use species beyond the genus for constructing a whole-genome sequence-based tree. Given that Brazil, located in South America, is widely acknowledged as the country of origin for water hyacinth, we opted to utilise a Brazilian species as the root taxon for our phylogenetic tree. This enables us to basically showcase the phylogenetic relationship between various lines. We have added the methods related to chloroplast genome assembly and other missing information in the revised version.

[Line 55: Can the authors explain a little bit about the taxonomy? Which name is accepted, which is synonym? If a formal taxonomic revision has been done, synonym name is not necessary here.]

>>Response: Thanks for your suggestions. Eichhornia crassipes (Mart.) Solms is as homotypic synonym of Pontederia crassipes Mart, which was revised in Taxonomy database of NCBI recently. Following your suggestion, we used Pontederia crassipes rather than Eichhornia crassipes in the revised version. However, the "Eichhornia crassipes" is the most commonly Latin name used in the current available scientific literature (several examples as followed). Regarding to consistency and clarity, we also provided the synonym in the context.

Monroy-Licht A, Méndez-Cuadro D, Olivero-Verbel J. Elemental mercury accumulation in Eichhornia crassipes (Mart.) Solms-Laubach. Environ Sci Pollut Res Int. 2023 Jan;30(4):9898-9913. doi: 10.1007/s11356-022-22521-y. Epub 2022 Sep 6. PMID: 36064851.
Feng W, Xiao K, Zhou W, Zhu D, Zhou Y, Yuan Y, Xiao N, Wan X, Hua Y, Zhao J. Analysis of utilization technologies for Eichhornia crassipes biomass harvested after restoration of wastewater. Bioresour Technol. 2017 Jan;223:287-295. doi: 10.1016/j.biortech.2016.10.047. Epub 2016 Oct 19. PMID: 27780621.

[Line 130: Phylogenetic position of E. crassipes?]

>>Response: Thank you for the above suggestions. We have modified the text accordingly.

[Line 131-137: This analysis with only eight species doesn't say much about the phylogeny of commelinids. To discuss the commelinids phylogeny, a proper sampling is needed, at least major clades in family level should be included.]

>>Response: Thanks for your suggestions. As mentioned in our manuscript, this is the first reference genome of the Commelinales and no genome of other families are available. We hope that the representative genome by this study will partially resolve the phylogenetic relationships of the Commelinales. Undoubtedly, we need more genomes of other families to fully resolve the phylogenetic relationships of the Commelinids in future.

[Line 131-137: The phylogenetic analysis is not clear. How did you construct the tree using 180 single-copy orthologs? Concatenated or coalescent? Information on alignment, model test, bootstrap etc. is missing.]

>>Response: Sorry for the confusion and we have added the missing information in the revised version. As mentioned above, this is the ultra-metric tree, transformed from the ML tree constructed based on concatenated sequence of 180 single-copy genes aligned by MAFFT. Bootstrap and model information about the ML tree have been provided in Supplementary Figure 3.
[Line 384: which database was used for BUSCO?]

>>Response: The database embryophyta_odb10 was used and we have added the information in the revised manuscript.

[Line 412: this is secondary calibration based on previous studies, rather than fossil constraint.]

>>Response: Thank you so much for your careful check. We have modified in the revised version.

[Figure 1A. Is this a concatenated or coalescent tree? Consensus or ML-best tree? Did you do bootstrap analysis on this?]

>>Response: Sorry for the inconvenience and we have added the information in the figure.

Reviewer #2:
[Line 43: I am not sure what is meant by "likely a result of fitness requirement for its role as a weed". What is a fitness requirement and a weedy habit have to do with each other? Please clarify.]

>>Response: Previous studies have revealed that the costs of resistance genes in plants. For example, the widely deployed mlo powdery mildew resistance gene in barley is associated with necrotic flecking and yield loss. Unlike crops, in which strong resistance is a necessary agronomic trait, weeds usually require rapid growth and massive reproduction. In this way, natural selection in weeds may favour reduced investment in resistance in order to maximize their reproductive capacity.

[Line 58: Please clarify if it is allo or auto polyploid here.]

>>Response: Sorry for the confusion. It’s an allotetraploid.

[Line 68:it is unclear from how this is written if rice, wheat and maize are specifically in the Commelinales or the more general commelinids clade with how this is written. Please specify "this clade" on line 69.]

>>Response: Thank you for pointing out this problem in the manuscript. We have re-written this part in the revised manuscript.

[Line 88: In general, the results had a lot of data interpretation and what I would consider discussion/conclusions. I suggest either having a single results and discussion section or being careful in the results when data is being interpreted and hypotheses are being presented.]

>>Response: Thank for your comments. We have adjusted the discussion section based on your comments and have moved some data interpretation in Results section to the Discussion section.

[Line 236-237: Highly speculative hypothesis. I would not say "likely" without some support or evidence. "Possibly" would be better. Better yet, maybe make it clear this is a hypothesis that needs testing.]

>>Response: Thanks for your suggestions. We have modified them in the revised version.

[Line 320: Could it also be gene loss due to no selection pressure in the maintainence of these genes? Genes will pseudogenize if they do not neo or sub functionalize and there is no selection to maintain them. It could be that these disease genes have low rates of neofunctionalization for one reason or another compared with other gene families. I would be extremely wary of prescribing a negative fitness cost when simply the lack of a positive fitness explains the same phenomenon. Also the point you bring on line 329-330 is also very good. Why maintain that which has no function; it should disappear due to drift.]

>>Response: Thank you for your rigorous comment. I’m agree with your speculation about the reasons of genes loss. In fact, there are multiple possibilities that could lead to a reduction in weed disease resistance genes. Fitness costs were found in a wide range of weeds, so we hypothesize that this is one explanation for this phenomenon

[Line 324: Clarify you mean pathogen resistant and not herbicide resistant in canary grass as we are talking about weeds.]

>>Response: We have re-written this part according to your comments.
