# Supplementary material for: A reference genome of Commelinales provides insights into the commelinids evolution and global spread of water hyacinth (Pontederia crassipes)
Source: Gigascience. 2024 Mar 14;13:giae006. doi: 10.1093/gigascience/giae006 (PMC10938897; doi:10.1093/gigascience/giae006)
Supplement: giae006_Reviewer_1_Report_Original_Submission — Eric Schranz -- 10/29/2023 Reviewed [file giae006_reviewer_1_report_original_submission.pdf]

Reviewer Report

Title: A reference genome of Commelinales provides insights into the commelinids evolution and global spread of water hyacinth (Pontederia crassipes)

Version: Original Submission **Date:** 10/29/2023

Reviewer name: Eric Schranz

Reviewer Comments to Author:

Major concerns:
1. The authors used 180 genes to infer the phylogeny of eight species, but the information about alignment, model test, bootstrap etc. are missing. It's not clear how did the authors generate the "orthologs-based tree". Did the author take it from OrthoFinder? Then the author built coalescent and concatenate trees and compared them with "orthologs-based tree". In general, both coalescent and concatenate trees are also orthologs-based tree. It seems that the authors compared the trees generated by different ML-based software (IQtree and RaxML) using the same dataset (180 orthologs). This doesn't say much about the robustness of the phylogeny but only to test the consistence between different software.
2. The authors included 10 lines to infer the global origin and dispersal of water hyacinth and concluded that "water hyacinth originates mainly from a single dispersal event from Brazil". Firstly, the sampling size is too small to derive such conclusion. Secondly, the chloroplast genomes are almost identical, with only a 1bp indel, which doesn't provide much information about phylo-geographic history. Thirdly, in the phylogeny (figure 4b), a Brazil line was put at the root. What's the justification? At least an outgroup is needed to root the tree. In addition, information about the chloroplast genome assembly, SNP calling, phylogeny inference etc. are missing.
Minor points:
Line 55: Can the authors explain a little bit about the taxonomy? Which name is accepted, which is synonym? If a formal taxonomic revision has been done, synonym name is not necessary here.
Line 130: Phylogenetic position of E. crassipes?
Line 131-137: This analysis with only eight species doesn't say much about the phylogeny of commelinids. To discuss the commelinids phylogeny, a proper sampling is needed, at least major clades in family level should be included.
Line 131-137: The phylogenetic analysis is not clear. How did you construct the tree using 180 single-copy orthologs? Concatenated or coalescent? Information on alignment, model test, bootstrap etc. is missing.
Line 384: which database was used for BUSCO?
Line 412: this is secondary calibration based on previous studies, rather than fossil constraint.
Figure 1A. Is this a concatenated or coalescent tree? Consensus or ML-best tree? Did you do bootstrap analysis on this?
Additional suggestions and comments that the authors could consider when revising their manuscript.
1. Legends for Supplementary Figures are missing from this submission? The downloaded separated

figures are without legends, therefore it was difficult to follow.
2. I understand that it was probably due to the way the superscaffolds were assembled and numbered from Hi-C data, however, would that be more consistent to rename superscaffolds in Figure S2 to match the numbers in Figure 1C? Now, it is up to superscaffold 19 (totally 16 superscaffolds, but some numbers in between are missing).
3. Also related to Figure S2, I assume that blue and green colors indicate the two subgenomes respectively. However, I think it would be helpful to state this somewhere in the manuscript or in the supplementary figure legends (currently missing).
4. For the 180 shared single-copy orthologs identified by Orthofinder among E. crassipes and seven other species, were whole genomes or sub-genomes used? I just wonder why there are just a few genes identified across the eight genomes. Also, this is not clearly described in the Methods where the authors described Orthofinder analysis.
5. Line 144: the authors wrote "the outcomes were also in accordance with a consensus tree generated using DensiTree [26] (Figure S3D)", however, the placement of M. balbisiana on the densitree (Figure S3D) is not consistent with that in Figures S3A-C. Could the authors clarify this?
6. A color scale for Figure S4 would be useful here. Does the color indicate syntenic relationship, e.g., red is highly syntenic?
7. Related to line 159 "pseudochromosomes into ancestral chromosomes as A1 (Chr1A ~ 4A), A2 (Chr5A ~ 8A), B1 (Chr1B ~ 4B) and B2 (Chr5B ~ 8B)", is this referring to Figure S4? Is Figure S4 a dotplot between 2 E. crassipes sub-genomes? If so, the chromosome names are the same for both axes (i.e., Ecra_1A vs Ecra_1A, instead of Ecra_1A vs Ecra_1B?). I found it is difficult to follow this clustering result. Would that be possible to illustrate it on the figure how the authors put the identified ancestral blocks together to make it 4 chromosomes, like a path or so?

Methods

Are the methods appropriate to the aims of the study, are they well described, and are necessary controls included? Choose an item.

Conclusions

Are the conclusions adequately supported by the data shown? Choose an item.

Reporting Standards

Does the manuscript adhere to the journal's guidelines on minimum standards of reporting? Choose an item.

Choose an item.

Statistics

Are you able to assess all statistics in the manuscript, including the appropriateness of statistical tests used? Choose an item.

Quality of Written English

Please indicate the quality of language in the manuscript: Choose an item.

Declaration of Competing Interests

Please complete a declaration of competing interests, considering the following questions:

- Have you in the past five years received reimbursements, fees, funding, or salary from an organisation that may in any way gain or lose financially from the publication of this manuscript, either now or in the future?
- Do you hold any stocks or shares in an organisation that may in any way gain or lose financially from the publication of this manuscript, either now or in the future?
- Do you hold or are you currently applying for any patents relating to the content of the manuscript?
- Have you received reimbursements, fees, funding, or salary from an organization that holds or has applied for patents relating to the content of the manuscript?
- Do you have any other financial competing interests?
- Do you have any non-financial competing interests in relation to this paper?

If you can answer no to all of the above, write 'I declare that I have no competing interests' below. If your reply is yes to any, please give details below.

'I declare that I have no competing interests'

I agree to the open peer review policy of the journal. I understand that my name will be included on my report to the authors and, if the manuscript is accepted for publication, my named report including any attachments I upload will be posted on the website along with the authors' responses. I agree for my report to be made available under an Open Access Creative Commons CC-BY license (http://creativecommons.org/licenses/by/4.0/). I understand that any comments which I do not wish to be included in my named report can be included as confidential comments to the editors, which will not be published.

Choose an item.

To further support our reviewers, we have joined with Publons, where you can gain additional credit to further highlight your hard work (see: https://publons.com/journal/530/gigascience). On publication of this paper, your review will be automatically added to Publons, you can then choose whether or not to claim your Publons credit. I understand this statement.

Yes Choose an item.
