# Supplementary material for: A reference genome of Commelinales provides insights into the commelinids evolution and global spread of water hyacinth (Pontederia crassipes)
Source: Gigascience. 2024 Mar 14;13:giae006. doi: 10.1093/gigascience/giae006 (PMC10938897; doi:10.1093/gigascience/giae006)
Supplement: giae006_Reviewer_2_Report_Original_Submission — Eric Patterson -- 11/1/2023 Reviewed [file giae006_reviewer_2_report_original_submission.pdf]

Reviewer Report

Title: A reference genome of Commelinales provides insights into the commelinids evolution and global spread of water hyacinth (Pontederia crassipes)

Version: Original Submission **Date:** 11/1/2023

Reviewer name: Eric Patterson

Reviewer Comments to Author:

A reference genome of Commelinales provides insights into the commelinids evolution
and global spread of water hyacinth (Eichhornia crassipes)
General Comments
I really, really like this paper and think the authors do an excellent job presenting a genome assembly in an interesting way and putting it fully. In its evolutionary conext. I also like that they use their assembly to resolve some outstanding phylogentic questions and add significantly to there clade. My only negative is their presentation of the hypothesis about disease resistance gene family contraction and its relationship with 'weediness'. I think the evidence for this relationship is very limited and should be deemphasized. If it were directly tested I would be happier, however, I find this to be speculative without further evidence. Everything else seems highly supported and very thorough.
Line Comments
Line 43: I am not sure what is meant by "likely a result of fitness requirement for its role as a weed". What is a fitness requirement and a weedy habit have to do with each other? Please clarify.
Line 58: Please clarify if it is allo or auto polyploid here.
Line 68:it is unclear from how this is written if rice, wheat and maize are specifically in the Commelinales or the more general commelinids clade with how this is written. Please specify "this clade" on line 69.
Line 88: In general, the results had a alot of data interpretation and what I would consider discussion/conclusions. I suggest either having a single results and discussion section or being careful in the results when data is being interpreted and hypotheses are being presented.
Line 236-237: Highly speculative hypothesis. I would not say "likely" without some support or evidence. "Possibly" would be better. Better yet, maybe make it clear this is a hypothesis that needs testing.
Line 320: Could it also be gene loss due to no selection pressure in the maintainence of these genes? Genes will pseudogenize if they do not neo or sub functionalize and there is no selection to maintain them. It could be that these disease genes have low rates of neofunctionalization for one reason or another compared with other gene families. I would be extremely wary of prescribing a negative fitness cost when simply the lack of a positive fitness explains the same phenomenon. Also the point you bring on line 329-330 is also very good. Why maintain that which has no function; it should disappear due to drift.
Line 324: Clarify you mean pathogen resistant and not herbicide resistant in canary grass as we are talking about weeds.

Methods

Are the methods appropriate to the aims of the study, are they well described, and are necessary controls included? Choose an item.

Conclusions

Are the conclusions adequately supported by the data shown? Choose an item.

Reporting Standards

Does the manuscript adhere to the journal's guidelines on minimum standards of reporting? Choose an item.

Choose an item.

Statistics

Are you able to assess all statistics in the manuscript, including the appropriateness of statistical tests used? Choose an item.

Quality of Written English

Please indicate the quality of language in the manuscript: Choose an item.

Declaration of Competing Interests

Please complete a declaration of competing interests, considering the following questions:

- Have you in the past five years received reimbursements, fees, funding, or salary from an organisation that may in any way gain or lose financially from the publication of this manuscript, either now or in the future?
- Do you hold any stocks or shares in an organisation that may in any way gain or lose financially from the publication of this manuscript, either now or in the future?
- Do you hold or are you currently applying for any patents relating to the content of the manuscript?
- Have you received reimbursements, fees, funding, or salary from an organization that holds or has applied for patents relating to the content of the manuscript?
- Do you have any other financial competing interests?
- Do you have any non-financial competing interests in relation to this paper?

If you can answer no to all of the above, write 'I declare that I have no competing interests' below. If your reply is yes to any, please give details below.

I declare that I have no competing interests. However, it should be noted that I recently began to mentor one of the middle co-authors, Eduardo Carlos Rudell, though I did not know this until after I accepted and completed by review.

I agree to the open peer review policy of the journal. I understand that my name will be included on my report to the authors and, if the manuscript is accepted for publication, my named report including any attachments I upload will be posted on the website along with the authors' responses. I agree for my report to be made available under an Open Access Creative Commons CC-BY license (http://creativecommons.org/licenses/by/4.0/). I understand that any comments which I do not wish to be included in my named report can be included as confidential comments to the editors, which will not be published.

Choose an item.

To further support our reviewers, we have joined with Publons, where you can gain additional credit to further highlight your hard work (see: https://publons.com/journal/530/gigascience). On publication of this paper, your review will be automatically added to Publons, you can then choose whether or not to claim your Publons credit. I understand this statement.

Yes Choose an item.
